# Supplementary figures and images for: Improved sleep, cognitive processing and enhanced learning and memory task accuracy with Yoga nidra practice in novices
Source: PLoS One. 2023 Dec 13;18(12):e0294678. doi: 10.1371/journal.pone.0294678 (PMC10718434; doi:10.1371/journal.pone.0294678)

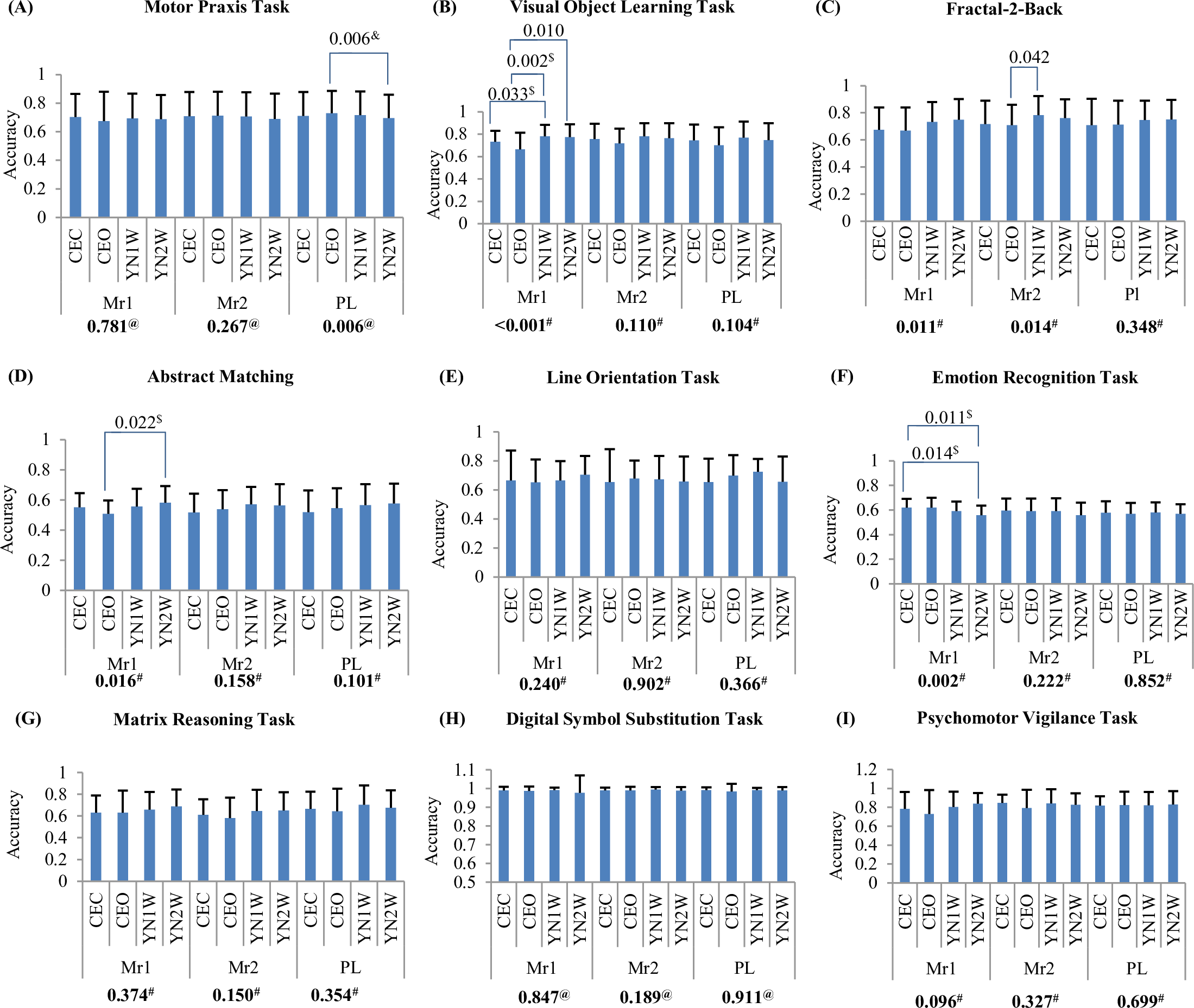

Supplement: S1 Fig — (Comparison model for each test condition i.e. CEC, CEO, YN1W, and YN2W is shown below each time i.e. Mr1, Mr2, and PL. Significant post-hoc p-values are also depicted) #: One-Way Repeated Measure (RM) Analysis of Variance (ANOVA) p-values $: Post-hoc analysis (pairwise comparison) is done using paired t-test with Bonferroni correction (significant changes indicated by p-values given above the bars). @: Friedman’s test p-value (if data is not normally distributed) &: Post-hoc analysis (pairwise comparison) is done using Wilcoxon Signed-Rank test with Bonferroni correction (significant changes indicated by p-values given above the bars). Mr1: Test just before CEC, CEO, YN1W or YN2W Mr2: Tests conducted just after CEO, C EC, YN1W or YN2W PL: Test conducted after the lunch CEO: Control with Eyes Open CEC: Control with Eyes Close YN1W: At the end of one week of Yoga Nidra practice after training YN2W: At the end of two weeks of Yoga Nidra practice after training. (TIF) [file pone.0294678.s001.tif]

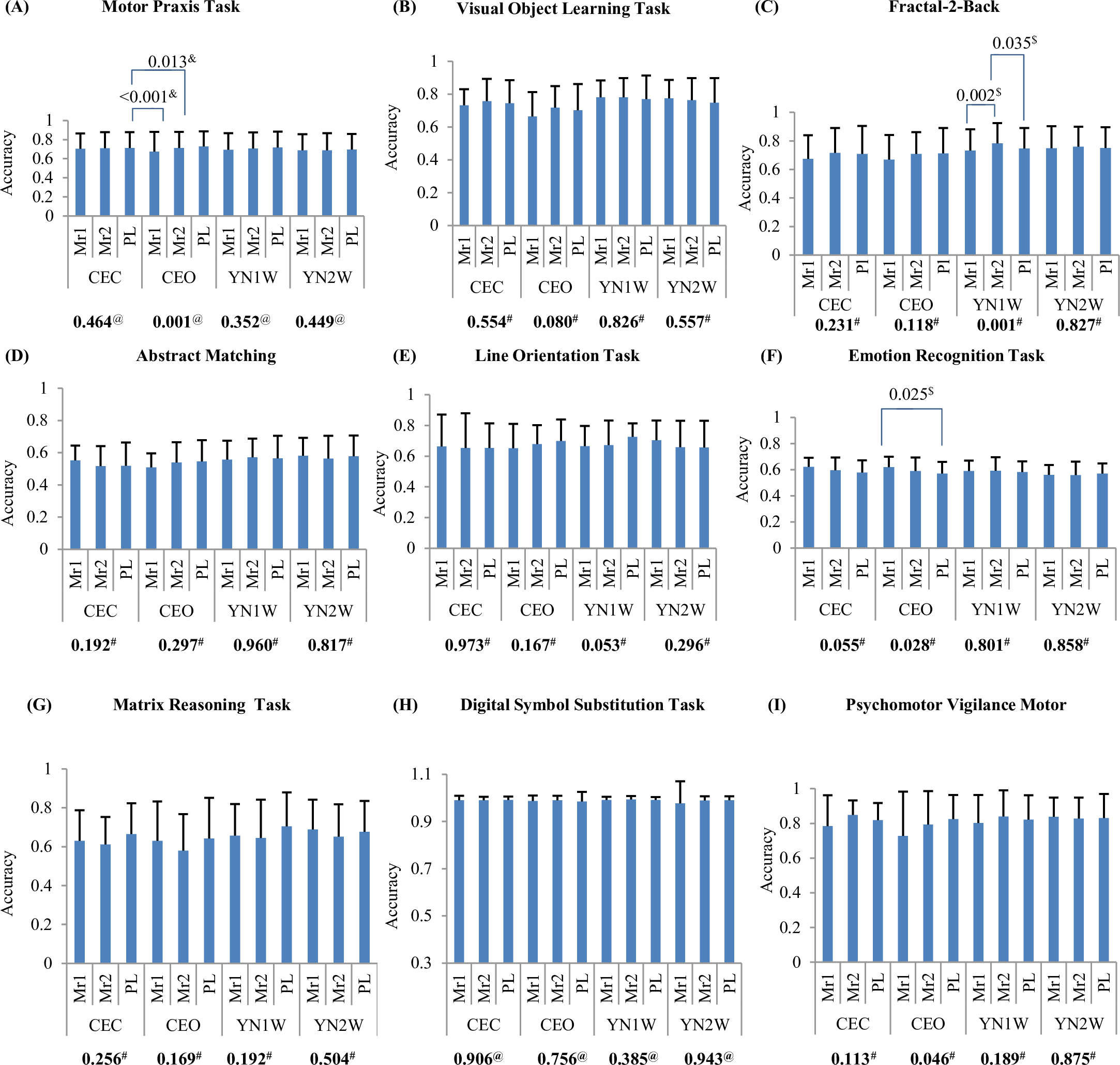

Supplement: S2 Fig — (Comparison model for each test condition i.e. CEC, CEO, YN1W, and YN2W is shown below each time i.e. Mr1, Mr2, and PL. Significant post-hoc p-values are also depicted) #: One-Way Repeated Measure (RM) Analysis of Variance (ANOVA) p-values $: Post-hoc analysis (pairwise comparison) is done using paired t-test with Bonferroni correction (significant changes indicated by p-values given above the bars). @: Friedman’s test p-value (if data is not normally distributed) &: Post-hoc analysis (pairwise comparison) is done using Wilcoxon Signed-Rank test with Bonferroni correction (significant changes indicated by p-values given above the bars). Mr1: Test just before CEC, CEO, YN1W or YN2W Mr2: Tests conducted just after CEO, C EC, YN1W or YN2W PL: Test conducted after the lunch CEO: Control with Eyes Open CEC: Control with Eyes Close YN1W: At the end of one week of Yoga Nidra practice after training YN2W: At the end of two weeks of Yoga Nidra practice after training. (TIF) [file pone.0294678.s002.tif]

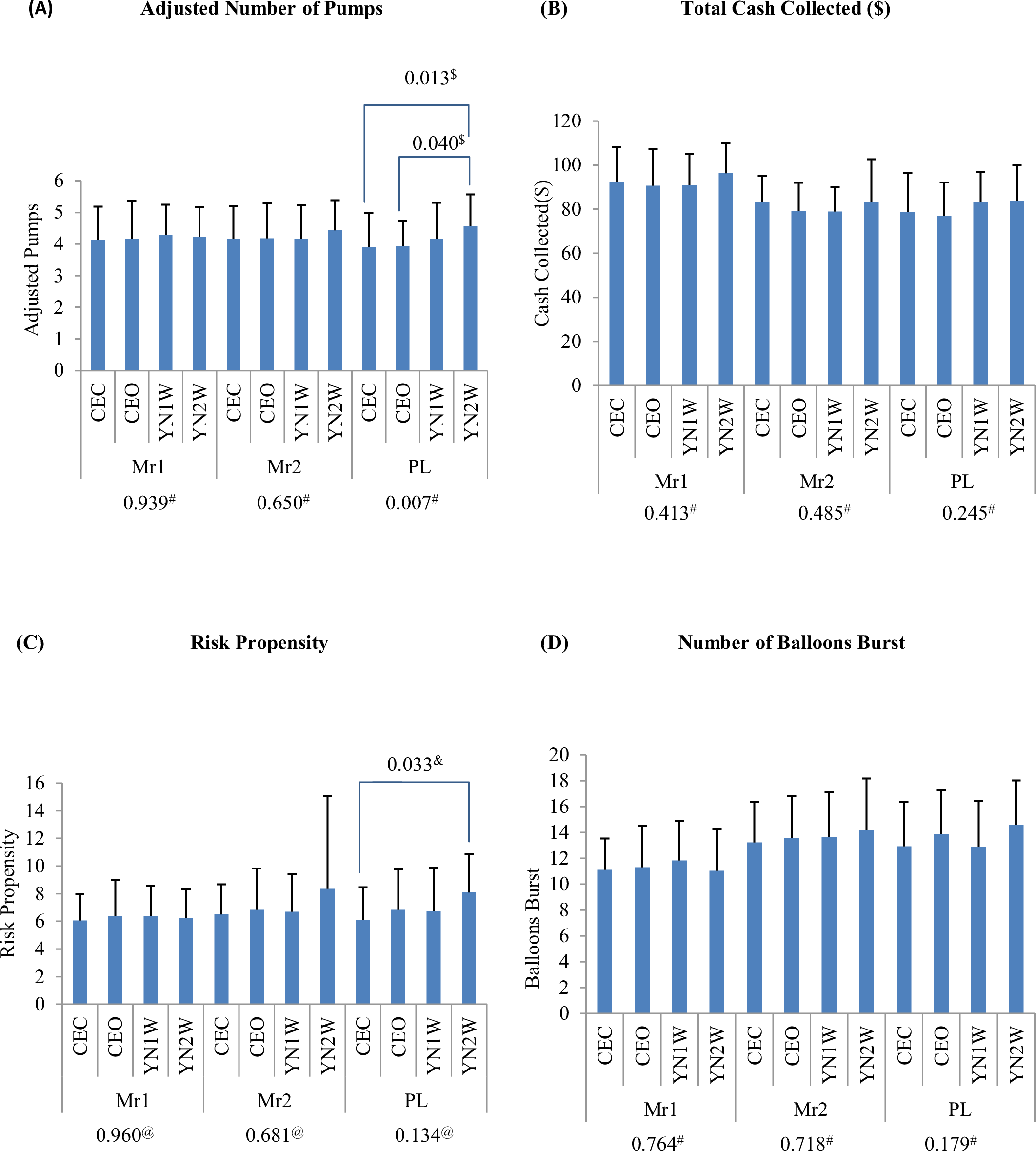

Supplement: S3 Fig — (P-values for time wise and test condition wise comparison model is shown below i.e. for Mr1, Mr2, and PL; and CEC, CEO, YN1W, and YN2W respectively. Significant post-hoc p-values are also depicted) Notes- #: One-Way Repeated Measure (RM) Analysis of Variance (ANOVA) p-values, $: Post-hoc analysis (pairwise comparison) is done using paired t-test with Bonferroni correction (significant changes indicated by p-values given above the bars)., @: Friedman’s test p-value (if data is not normally distributed), &: Post-hoc analysis (pairwise comparison) is done using Wilcoxon Signed-Rank test with Bonferroni correction (significant changes indicated by p-values given above the bars). Mr1: Test just before CEC, CEO, YN1W or YN2W Mr2: Tests conducted just after CEO, C EC, YN1W or YN2W PL: Test conducted after the lunch CEO: Control with Eyes Open CEC: Control with Eyes Close YN1W: At the end of one week of Yoga Nidra practice after training YN2W: At the end of two weeks of Yoga Nidra practice after training. (TIF) [file pone.0294678.s003.tif]

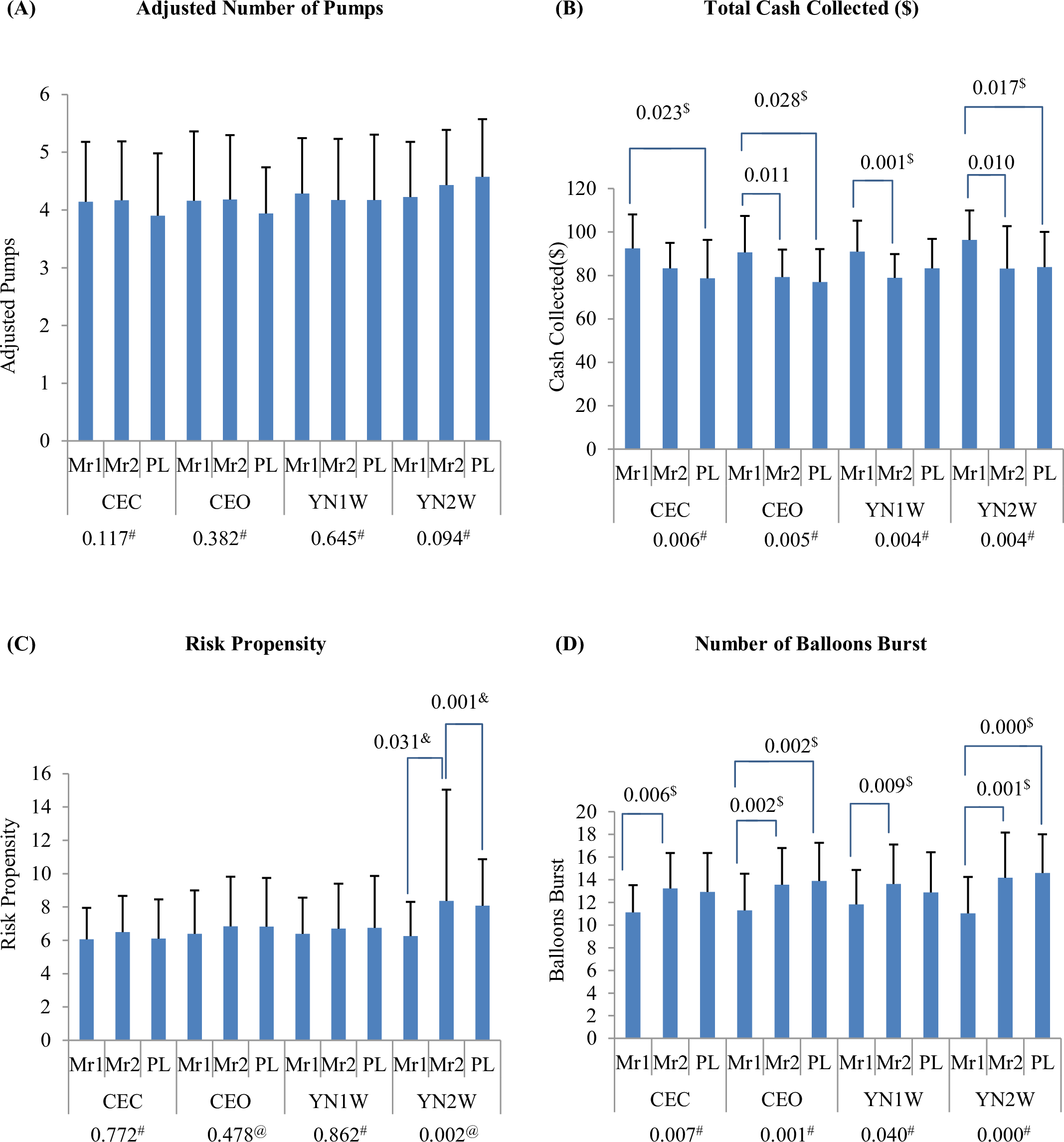

Supplement: S4 Fig — (P-values for time wise and test condition wise comparison model is shown below i.e., for Mr1, Mr2, and PL; and CEC, CEO, YN1W, and YN2W respectively. Significant post-hoc p-values are also depicted). Notes- #: One-Way Repeated Measure (RM) Analysis of Variance (ANOVA) p-values, $: Post-hoc analysis (pairwise comparison) is done using paired t-test with Bonferroni correction (significant changes indicated by p-values given above the bars)., @: Friedman’s test p-value (if data is not normally distributed), &: Post-hoc analysis (pairwise comparison) is done using Wilcoxon Signed-Rank test with Bonferroni correction (significant changes indicated by p-values given above the bars). Mr1: Test just before CEC, CEO, YN1W or YN2W Mr2: Tests conducted just after CEO, C EC, YN1W or YN2W. PL: Test conducted after the lunch CEO: Control with Eyes Open. CEC: Control with Eyes Close YN1W: At the end of one week of Yoga Nidra practice after training. YN2W: At the end of two weeks of Yoga Nidra practice after training. (TIF) [file pone.0294678.s004.tif]

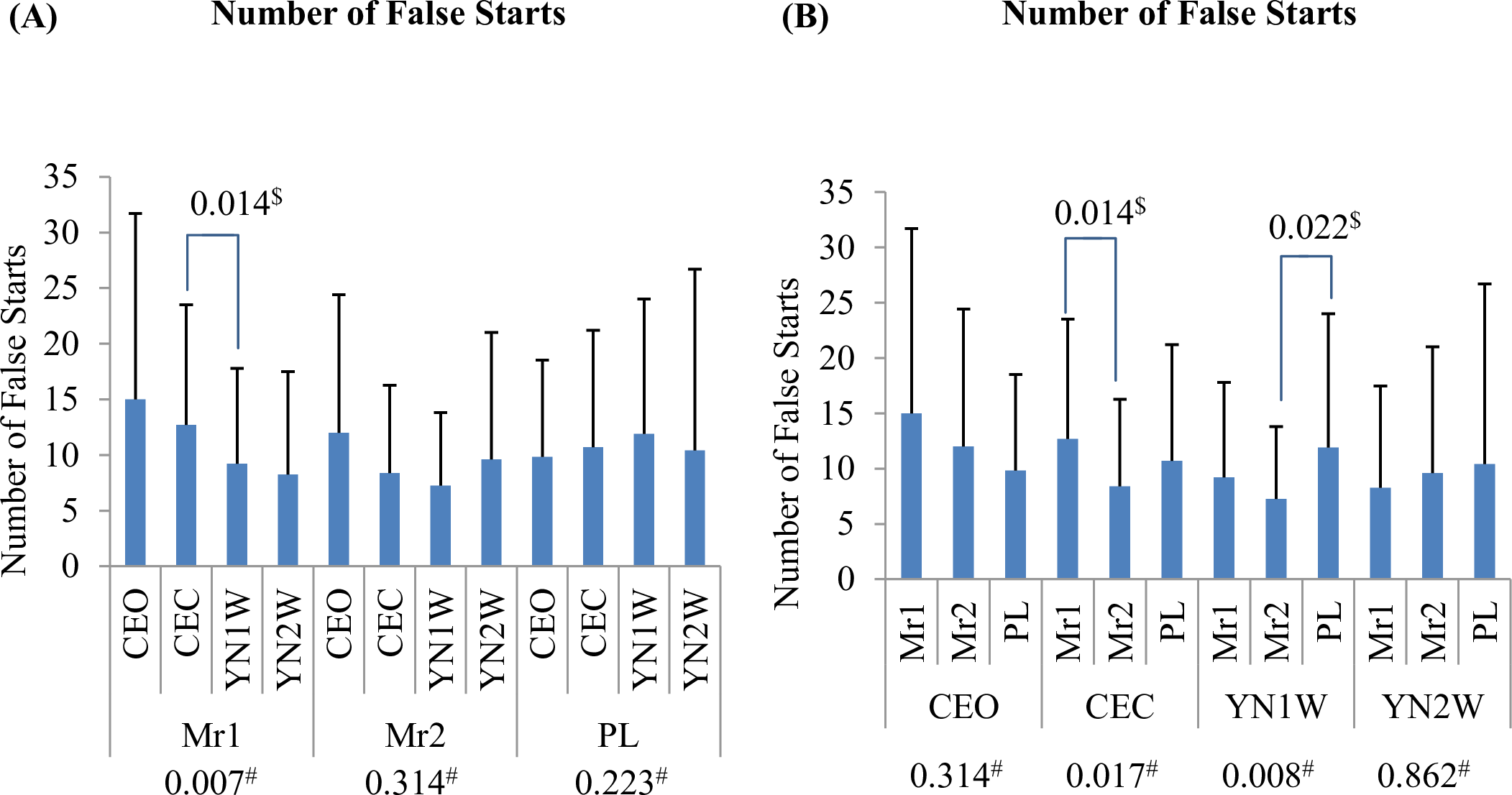

Supplement: S5 Fig — Comparison model for each test condition i.e. CEC, CEO, YN1W, and YN2W is shown below each time i.e. Mr1, Mr2, and PL. Significant post-hoc p-values are also depicted. (TIF) [file pone.0294678.s005.tif]
