# Supplementary material for: Improved sleep, cognitive processing and enhanced learning and memory task accuracy with Yoga nidra practice in novices
Source: PLoS One. 2023 Dec 13;18(12):e0294678. doi: 10.1371/journal.pone.0294678 (PMC10718434; doi:10.1371/journal.pone.0294678)
Supplement: Attachment — Submitted filename: PONE-D-23-21201 Rev.pdf [file pone.0294678.s010.pdf]

PLOS ONE

Improved Sleep, Cognitive Processing and Enhanced Learning and Memory Task Accuracy with Yoga Nidra Practice in Novices

--Manuscript Draft--

**Manuscript Number:**	PONE-D-23-21201
**Article Type:**	Research Article
**Full Title:**	Improved Sleep, Cognitive Processing and Enhanced Learning and Memory Task Accuracy with Yoga Nidra Practice in Novices
**Short Title:**	Improved sleep and learning-memory after yoga nidra practice
**Corresponding Author:**	Karuna Datta, M.D., Ph.D.<br>Armed Forces Medical College<br>pune, INDIA
**Keywords:**	yoga nidra; cognitive function; polysomnography; EEG [illegible] [illegible]y; sleep health
**Abstract:**	Complementary and Alternative medicine has its own benefits. Yoga nidra practice is an easy-to-do practice and has shown beneficial effects on stress reduction. It has also been found to improve sleep in insomnia patients. Effect of yoga nidra practice on subjective sleep is known but its effect on sleep and cognition objectively is not documented. The aim of the study was to study the effect of yoga nidra practice on cognition and sleep using objective parameters. 41 participants were enrolled, and baseline sleep diary (SD) collected. Participants volunteered for overnight polysomnography (PSG) and cognition testing battery (CTB) comprising of Motor praxis test, emotion recognition task (ERT), digital symbol substitution task, visual object learning task (VOLT), abstract matching (AIM), line orientation task , matrix reasoning task , fractal-2-back test (NBACK), psychomotor vigilance task and balloon analog risk task . Baseline CTB and after one and two weeks of practice was compared. Power spectra density for EEG at central, frontal, and occipital locations during CTB was compared. Repeat SD and PSG after four weeks of practice were done. After yoga nidra practice, improved reaction times for all cognition tasks, Post intervention compared to baseline (95%CI; p-value) was +3.62% (0.3, 5.15; p-value=0.03) , -20min (-35.78, -5.02; p=0.003) and +4.19 (0.5, 9.5; p=0.04) in sleep efficiency, wake after sleep onset and delta µV2 in deep sleep respectively. Accuracy increased in VOLT (95% CI: 0.08, 0.17; p=0.002), AIM (95% CI: 0.03, 0.12; p= 0.02) and NBACK (95% CI: 0.02, 0.13; p=0.04); ERT accuracy increased for happy, fear and anger (95% CI: 0.07, 0.24; p=0.004) but reduced for neutral stimuli (95% CI: -0.3, -0.05; p=0.04) after yoga nidra practice. Yoga nidra practice improved cognitive processing and night-time sleep.
**Order of Authors:**	Karuna Datta, M.D., Ph.D.
	Anna Bhutambare, M.sc.
	Mamatha VL, MCA
	Yogita Narawade, M.Sc
	Srinath Rajagopal, MD, DM
	Madhuri Kanitkar, MD
**Additional Information:**	
**Question**	**Response**
**Financial Disclosure**<br><br>Enter a financial disclosure statement that describes the sources of funding for the work included in this submission. Review the submission guidelines for detailed	KD received the funding from Department of Science and Technology under Science and Technology for Yoga and Meditation (SATYAM), India available at https://dst.gov.in/, and was received vide their sanction order DST Satyam 2018/457 dated 28 May 2020.The funders had no role in study design, data collection and analysis, decision to publish, or preparation of the manuscript.

compared to what

requirements. View published research articles from *PLOS ONE* for specific examples.<br><br>This statement is required for submission and **will appear in the published article** if the submission is accepted. Please make sure it is accurate.<br><br>**Unfunded studies**<br>Enter: *The author(s) received no specific funding for this work.*<br><br>**Funded studies**<br>Enter a statement with the following details:<br>• Initials of the authors who received each award<br>• Grant numbers awarded to each author<br>• The full name of each funder<br>• URL of each funder website<br>• Did the sponsors or funders play any role in the study design, data collection and analysis, decision to publish, or preparation of the manuscript?<br>• **NO** - Include this sentence at the end of your statement: *The funders had no role in study design, data collection and analysis, decision to publish, or preparation of the manuscript.*<br>• **YES** - Specify the role(s) played.<br><br>* typeset	
**Competing Interests**<br><br>Use the instructions below to enter a competing interest statement for this submission. On behalf of all authors, disclose any competing interests that could be perceived to bias this work—acknowledging all financial support and any other relevant financial or non-financial competing interests.<br><br>This statement is required for submission and **will appear in the published article** if the submission is accepted. Please make sure it is accurate and that any funding sources listed in your Funding Information later in the submission form are also declared in your Financial Disclosure	The authors have declared that no competing interests exist.

Powered by Editorial Manager® and ProduXion Manager® from Aries Systems Corporation

statement.<br><br>View published research articles from *PLOS ONE* for specific examples.<br><br>**NO authors have competing interests**<br><br>Enter: *The authors have declared that no competing interests exist.*<br><br>**Authors with competing interests**<br><br>Enter competing interest details beginning with this statement:<br><br>*I have read the journal's policy and the authors of this manuscript have the following competing interests: [insert competing interests here]*<br><br>* typeset	
**Ethics Statement**<br><br>Enter an ethics statement for this submission. This statement is required if the study involved:<br><br>• Human participants<br>• Human specimens or tissue<br>• Vertebrate animals or cephalopods<br>• Vertebrate embryos or tissues<br>• Field research<br><br>Write "N/A" if the submission does not require an ethics statement.<br><br>General guidance is provided below. Consult the submission guidelines for detailed instructions. **Make sure that all information entered here is included in the Methods section of the manuscript.**	Armed Forces Medical College (IEC/2019/255 dated 25 Apr 2019). The study was registered in CTRI.in, vide no. CTRI/2021/031349.<br>Written informed consent was taken.

Powered by Editorial Manager® and ProduXion Manager® from Aries Systems Corporation

**Format for specific study types**<br><br>**Human Subject Research (involving human participants and/or tissue)**<br>• Give the name of the institutional review board or ethics committee that approved the study<br>• Include the approval number and/or a statement indicating approval of this research<br>• Indicate the form of consent obtained (written/oral) or the reason that consent was not obtained (e.g. the data were analyzed anonymously)<br><br>**Animal Research (involving vertebrate animals, embryos or tissues)**<br>• Provide the name of the Institutional Animal Care and Use Committee (IACUC) or other relevant ethics board that reviewed the study protocol, and indicate whether they approved this research or granted a formal waiver of ethical approval<br>• Include an approval number if one was obtained<br>• If the study involved *non-human primates*, add *additional details* about animal welfare and steps taken to ameliorate suffering<br>• If anesthesia, euthanasia, or any kind of animal sacrifice is part of the study, include briefly which substances and/or methods were applied<br><br>**Field Research**<br><br>Include the following details if this study involves the collection of plant, animal, or other materials from a natural setting:<br>• Field permit number<br>• Name of the institution or relevant body that granted permission	
**Data Availability**<br><br>Authors are required to make all data underlying the findings described fully available, without restriction, and from the time of publication. PLOS allows rare exceptions to address legal and ethical concerns. See the PLOS Data Policy and FAQ for detailed information.	Yes - all data are fully available without restriction

A Data Availability Statement describing where the data can be found is required at submission. Your answers to this question constitute the Data Availability Statement and **will be published in the article**, if accepted.<br><br>**Important:** Stating 'data available on request from the author' is not sufficient. If your data are only available upon request, select 'No' for the first question and explain your exceptional situation in the text box.<br><br>Do the authors confirm that all data underlying the findings described in their manuscript are fully available without restriction?	
**Describe where the data may be found in full sentences. If you are copying our sample text, replace any instances of XXX with the appropriate details.**<br><br>• If the data are **held or will be held in a public repository**, include URLs, accession numbers or DOIs. If this information will only be available after acceptance, indicate this by ticking the box below. For example: *All XXX files are available from the XXX database (accession number(s) XXX, XXX.).*<br>• If the data are all contained **within the manuscript and/or Supporting Information files**, enter the following: *All relevant data are within the manuscript and its Supporting Information files.*<br>• If neither of these applies but you are able to provide **details of access elsewhere**, with or without limitations, please do so. For example:<br><br>*Data cannot be shared publicly because of [XXX]. Data are available from the XXX Institutional Data Access / Ethics Committee (contact via XXX) for researchers who meet the criteria for access to confidential data.*<br><br>*The data underlying the results presented in the study are available from (include the name of the third party*	All relevant data are within the manuscript and its Supporting Information files.

and contact information or URL).<br>• This text is appropriate if the data are owned by a third party and authors do not have permission to share the data.<br><br>* typeset	
Additional data availability information:	

Manuscript

Click here to access/download;Manuscript;14 Jul Plos one main.docx

Title Page

Title of the article: - ***Improved Sleep, Cognitive Processing and Enhanced Learning and Memory Task Accuracy with Yoga Nidra Practice in Novices***

Karuna Datta (Corresponding Author)
Human Sleep Research Lab. c/o Department of Sports Medicine
Armed Forces Medical College
Pune, Maharashtra, India.
Email: karunadatta@gmail.com
Phone-+91-9560118881

Anna Bhutambare
JRF, DST SATYAM project, Human Sleep Research Lab
c/o Department of Sports Medicine
Armed Forces Medical College
Pune, Maharashtra, India.
Mamatha VL
Research Associate, DST SATYAM project, Human Sleep Research Lab
c/o Department of Sports Medicine
Armed Forces Medical College
Pune, Maharashtra, India.
Yogita Narawade
JRF, DST SATYAM project, Human Sleep Research Lab
c/o Department of Sports Medicine
Armed Forces Medical College
Pune, Maharashtra, India.
Srinath Rajagopal
Department of Internal Medicine
Armed Forces Medical College
Pune, Maharashtra, India.
Madhuri Kanitkar
Vice Chancellor, Maharashtra University of Health Sciences, Nashik
Maharashtra, India

Keywords: yoga nidra; cognitive function; polysomnography; EEG power spectra density; sleep health

Abstract:

Complementary and Alternative medicine has its own benefits. Yoga nidra practice is an easy-to-do practice and has shown beneficial effects on stress reduction. It has also been found to improve sleep in insomnia patients. Effect of yoga nidra practice on subjective sleep is known but its effect on sleep and cognition objectively is not documented. The aim of the study was to study the effect of yoga nidra practice on cognition and sleep using objective parameters. 41 participants were enrolled, and baseline sleep diary (SD) collected. Participants volunteered for overnight polysomnography (PSG) and cognition testing battery (CTB) comprising of Motor praxis test, emotion recognition task (ERT), digital symbol substitution task, visual object learning task (VOLT), abstract matching (AIM), line orientation task , matrix reasoning task , fractal-2-back test (NBACK), psychomotor vigilance task and balloon analog risk task . Baseline CTB and after one

and two weeks of practice was compared. Power spectra density for EEG at central, frontal, and occipital locations during CTB was compared. Repeat SD and PSG after four weeks of practice were done. After yoga nidra practice, improved reaction times for all cognition tasks, Post intervention compared to baseline (95%CI; p-value) was +3.62% (0.3, 5.15; p-value=0.03) , -20min (-35.78, -5.02; p=0.003) and +4.19 (0.5, 9.5; p=0.04) in sleep efficiency, wake after sleep onset and delta μV^2 in deep sleep respectively. Accuracy increased in VOLT (95% CI: 0.08, 0.17; p=0.002), AIM (95% CI: 0.03, 0.12; p= 0.02) and NBACK (95% CI: 0.02, 0.13; p=0.04); ERT accuracy increased for happy, fear and anger (95% CI: 0.07, 0.24; p=0.004) but reduced for neutral stimuli (95% CI: -0.3, -0.05; p=0.04) after yoga nidra practice. Yoga nidra practice improved cognitive processing and night-time sleep.

1. Introduction:

Complementary and Alternative medicine has its own benefits in both health and disease(1). A concept of therapeutic yoga thus emerged, and a need to build standardised models became vital. Though available studies on yoga have different methodologies(2), but the ease of doing yoga practice makes it more approachable by healthy population(3). If an easy practice can be made as a standardised model for learning it, thus improving its effectiveness, it can be promoted for wellbeing in population studies.

Yoga nidra practice, a kind of *pratyahara* (withdrawal of senses) technique, is an easy-to-do practice(4). It has been described as an ‘awake aware sleep’ which is different from meditation and hypnosis (4,5). It has been associated with stress reduction, and has been used for patients of insomnia, menstrual abnormalities, post-traumatic stress disorder, etc and also for sportsperson [illegible] Yoga nidra practice has been standardised for learning by novices(12), thus improving the [illegible]

Good sleep quality is essential for human [illegible] decisions require optimized cognitive functioning. There [illegible]nitive functioning whereas partial sleep deprivation reduces it (1[illegible]

> Yoga Nidra can also be defined as certain form of mindfulness meditation; please make clear what you mean when claiming it as

Research literature of effects of yoga nidra in meditators has been published widely (14–17) with an evidence of a presence of distinct state, different from meditation. These subjects being long term practitioners cannot be compared to novices who are still learning the practice and are not trained meditators too. Moreover, the effects of yoga nidra in long-term meditators are also because of the effects of meditation. Thus, there is a need to study effects of yoga nidra on novices to understand its use in population. The methodology for yoga nidra practice by novices is already been laid out(4). Yoga nidra is proven to improve sleep in insomnia patients(6,12). Subjective improvement of night-time sleep in healthy novices has been reported (7,18,19) and recently, an evidence of local sleep during morning yoga nidra practice has also been seen(18). However its effect on objective parameters of sleep in healthy novices is not documented. It is also not known if yoga nidra practice improves cognitive performance. There was thus a felt need to study the effect of yoga nidra practice by novices on their sleep and cognition objectively.

2. Methods

2.1 Study Design and Participants:

The project was a pre-post interventional cohort study with an aim to study the effect of yoga nidra practice on cognitive function and sleep.

The study was conducted on healthy volunteers and their cognition was assessed at baseline and after yoga nidra practice. Objective sleep parameters using overnight polysomnography (PSG) were also studied at baseline and after yoga nidra practice.

Yoga nidra supervised training model for novices developed by Datta et al ,(6,12) was used to study the effects of yoga nidra practice on cognitive function and on night sleep. Participants were assessed after one and two weeks of practicing yoga nidra. Details of study design are given in **Fig 1(A) and Fig 1(B)** for both objectives, i.e., the effect of yoga nidra practice on cognitive function and on night-time objective sleep parameters respectively.

Fig 1: Study Design for Effect of Yoga Nidra Practice on Cognitive Function (**Fig 1A**) and on Objective Sleep Parameters (**Fig 1B**)

The study was conducted during 2021-2022 after institutional ethical clearance (IEC/2019/255 dated 25 Apr 2019). The study was registered in CTRI.in, vide no. CTRI/2021/031349. Local advertisements in the city were placed and volunteers a[illegible]ch of the objectives were considered for th[illegible]

Please explain thoroughly why you decided to exclude women and discuss its limitations to do so

2.2 Screening of Particip[illegible]

Healthy young male volunteers with a consistent sleep wake schedule were recruited. All participants underwent a thorough examination by a medical practitioner. Sleep wake schedule was monitored, and a baseline sleep diary of 14 days obtained. Exclusion criteria included any sleep disorder, depression, h/o psychiatric disorder, and h/o acute illness which were likely to affect sleep wake schedule.

2.3 Intervention: Yoga-Nidra Practice

A pre-recorded yoga nidra audio was used (available *at https://youtu.be/0fmMlELn-Ug*). Each Yoga-Nidra session consisted of seven steps, namely: preparation, *samkalpa* (resolution), body part awareness, breath awareness, feeling and sensation, visualization, and ending of practice. Initially, yoga nidra training was done using supervised Yoga-Nidra sessions as per the therapeutic model developed earlier. During these supervised sessions, the investigator followed the ‘guidelines for observer’ developed for practice(6,12).. The participants continued the practice on their own after the supervised training was done. Follow up testing was done on participants as per the study design given in **Fig 1(A) and Fig 1(B).**

2.4 Sleep Diary (SD) Analysis:

All participants filled a baseline sleep diary for 14 days. The sleep diary was filled twice a day, once in the morning just after getting up and again at night just before bedtime. The participants continued to fill the sleep diary during the intervention. The sleep diary parameters obtained were: time in bed (sdTIB), total sleep time (sdTST), sleep onset latency (sdSOL), wake after sleep onset (sdWASO), sleep quality (sdSQ), and sleep efficiency (sdSE)(12). These parameters were extracted from the two-week data of baseline and post-intervention, i.e. Two weeks diary after four weeks of yoga nidra practice.

Data Analysis: Sleep diary data was analyzed for pre and post comparisons. Normality of data was checked using Shapiro-Wilks test. If data was normally distributed, paired t-test was used to compare sleep diary parameters before (baseline) yoga nidra practice and after (post-intervention) yoga nidra practice. For non-normal data several transformations like square, square root, log, and exponential were tried. If normality was

not achieved, the Wilcoxon signed rank test was used. Statistical significance was considered at alpha 0.05. All statistical analyses were done using R-software (version 4.1.2).

2.5 Methodology for assessing the effect of yoga nidra practice on cognitive function.

The cognition test battery (CTB) was employed using the Joggle Research Platform (USA). Our battery consisted of ten tests in a standard sequence of motor praxis task (MPT), visual object learning task (VOLT), fractal-2-back(NBACK), abstract matching(AIM), line orientation task(LOT), emotion recognition task(ERT), matrix reasoning task(MRT), digital symbol substitute task(DSST), balloon analog risk task(BART), and psychomotor vigilance task (PVT:10min). The various cognition domains that were assessed by these 10 tests were: MPT – sensory motor speed, VOLT – spatial learning and memory, NBACK – working memory, AIM – abstraction and concept information, LOT – spatial orientation, ERT – emotion identification, MRT – abstract reasoning, DSST – complex scanning and visual tracking, BART – risk decision making, and PVT – vigilant attention(20).

CTB was performed with the test conditions of control with eyes open (CEO), control with eyes close (CEC), at the end of 1st week of yoga nidra practice (YN1W), and at the end of 2nd week of yoga nidra practice (YN2W). All the participants completed the CTB tests three times on each test condition day i.e., at 1000h (Mr1), immediately after the test condition of CEC, CEO, YN1W, or YN2W (Mr2) and then in the afternoon at 1500h (post lunch: PL). The conditions were kept standardized by similar food, working environment on all condition days for each participant. Detailed representation of study design is shown in **Fig 1(A).**

CTB outcome variables studied were reaction time (for all 10 tests) and accuracy score for 9 tests, except BART. For BART –'Adjusted Number of Pumps', 'Number of Explosions', 'Risk Propensity' and 'Cash collected' were analyzed(21). 'Adjusted Pumps' is the average number of pumps on each balloon during BART when the balloon did not burst, 'Number of Explosions' is the total number of balloons exploded during BART, 'Risk Propensity' is the ratio of total pumps/balloons collected, and 'Cash collected' is the amount of reward earned in BART (20). The standard accuracy outcome was 'proportion correct' ranging from 'zero' to 'one', where 'zero' and 'one' represented worst and best possible performance respectively. For the MPT, the distance from the center of the square to the point where participant touches was used as an accuracy score. The center of square translates to 'one' accuracy score and an edge of square to 'zero' accuracy score, with linear scaling between center and edges. For LOT, the accuracy score was calculated as 'three' minus the average number of clicks off, which was then divided by 'three'. For tests with average number of clicks off is more than 'three', the accuracy score was set to 'zero'. For PVT, the accuracy score was calculated as 'one' – [(number of lapses + number of false starts) / (number of stimuli + number of false starts)]. PVT- number of false starts was analyzed apart from reaction time and accuracy(20). ERT was also studied for various types of stimuli apart from reaction time and accuracy.

Data Analysis: One-Way Repeated Measures (RM) Analysis of Variance (ANOVA) for checking significant change in each of the outcome variable between different test conditions (CEO, CEC, YN1W, and YN2W) and at different times (Mr1, Mr2, or PL) was used for normal data. If the data was not normal, Friedman's test was used. In instances where the sphericity assumption was not met, the reported p-values associated with the F-statistics were adjusted via the Greenhouse-Geisser correction. If the ANOVA model was significant, post-hoc analysis (pair wise comparison) was done using paired t-test with Bonferroni Correction. Wilcoxon Signed Rank Test with Bonferroni Correction was used for post-hoc analysis (pair wise comparison) if Friedman's Test was significant. Bonferroni Correction was used to reduce the probability of type-1 error. All

reported p-values were two-tailed and statistical significance was assumed if p-value < 0.05. All statistical analyses were done using R (version 4.1.2).

2.6 Power spectral density (PSD) of EEG daytime recording during CTB:

The power spectral analysis of EEG data was performed using MNE-Python, an open-source library for visualizing, analyzing and exploring the raw EEG signal(22). PSD analysis was planned out for the EEG data of 12 participants who volunteered for the EEG acquisition during CTB. This included loading data, pre-processing, and segmentation of the continual data on the obtained epoched data. Time-Frequency investigation was performed and finally spectral-parameters of the EEG signal was extracted (23). Spectral investigation was based on frequency bands. PSD is the reflection of frequency components of EEG signal(24). Preprocessing included low and high frequency filtering of the EEG signal, notch filtering and removal of bad segments of the data (made by visual screening of data for sudden artifacts). Eye movements were repaired using the artifacts repair regression technique, custom re-referencing, and segmentation of the EEG data to epochs. Filtering was done at low-frequency (1Hz), high-frequency (90Hz) and notch-filter at 50Hz. After manual marking of artifacts and bad segments, MNE-Python library was used to automatically exclude annotated spans of data while creating the epochs from the continual EEG data. Special care was taken to avoid segments with artifacts or noise so that artifact free data would be extracted for further analysis. The EEG signal acquisition was based on the domain of time and future extraction. Slow eye movement artifacts in the EEG were repaired using computed coefficients(25).

Segmentation was performed on the clean raw EEG data to segment the EEG data to epochs. 'Feature extraction' was done based on 'Time-Frequency domain' (23),(26). The time-frequency domain depicts the distribution of power of the EEG signal with respect to Time-Frequency plane. The computation of PSD was carried out using Welch's Technique. The decomposition of signal to frequency bands were done using Fourier transforms (23). The ranges of the bands were delta (1-4Hz), theta1 (4-6Hz), theta2 (6-8Hz), alpha1 (8-10Hz), alpha2 (10-12.5Hz) and beta (13-30Hz)(18). The features used in the EEG signal analysis were relative-band-power and ratios between the bands (27). On each test segment spectral analysis was performed for all the EEG channels. Using 256Hz sampling rate of EEG, feature extraction was done from the desired test segments for all the EEG channels on every individual participant using 512ms window duration. The specified spectral analysis methodology was employed for all the participants on all test conditions i.e., CEC, CEO, YN1W, and YN2W for all the times i.e., Mr1, Mr2, and PL. PSD values were compared for various frequency bands and as ratios. The various parameters thus studied were PSD values of delta, theta1, theta2, alpha1, alpha2, beta and ratios of delta/beta, theta1/beta, theta2/beta, theta1/alpha1, theta2/alpha1, theta1/alpha2, theta2/alpha2, delta/theta1, delta/theta2, delta/alpha1, and delta/alpha2.

Data Analysis: One-way ANOVA model was used for checking significance of difference in the PSD outcome parameters and post-hoc analysis using Tukey post-hoc test was done, if the data was normal. Otherwise Kruskal Wallis test was used with Wilcoxon test for post-hoc analysis. Statistical significance was considered at alpha 0.05. All statistical analyses were done using R (version 4.1.2).

2.7 Methodology for assessing the effect of yoga nidra practice on objective sleep parameters.

Overnight PSG was done in 30 participants to assess the effect of yoga nidra on objective sleep parameters. PSG was conducted according to AASM criteria(28) using 'SOMNOMEDICS©, Germany PSG System'. EEG, EOG, EMG channels were placed for scoring sleep-wake stages using DOMINO© software version

3.0.0.6. F3, F4, C3, C4, O1, and O2 EEG channels were placed as per the 10-20 system. PSG study was done during the night under standard conditions. The impedance was tried to be kept below 5KΩ. PSG was performed twice, at baseline (BL PSG) and post-intervention i.e., after four weeks of yoga nidra practice after initial training (PI PSG).

PSG data files were analyzed by KD in groups of 15-20 files and each was coded to blind KD from the identity state of subject data i.e., of baseline (BL) or post-intervention (PI). The files once analyzed were decoded by AB for further analysis.

Various parameters obtained using PSG were time in bed (TIB), total sleep time (TST), wake duration, wake after sleep onset (WASO), and duration of various stages of sleep (i.e., N1, N2, N3 and REM). Amplitude of alpha, beta, theta and delta waves was recorded in μV^2. Percentages for sleep, wake, REM, and Non-REM sleep were calculated and analyzed.

Data Analysis: Normality of data was checked using Shapiro-Wilk's test for normality. Quantile-Quantile plot for each of the outcome parameter was made. If normality was not obtained even after square root, square, log, and exponential transformations then analysis was done using Wilcoxon Signed Rank Test. All statistical analyses were done using R (version 4.1.2).

3. Results and Discussion

Forty-one participants were enrolled in the study. Details of number of participants screened, allocated, and finally analyzed are shown in **Fig 2**. Some participants could not report for testing due to the COVID pandemic during the time of study, though they continued the intervention. No adverse effects were reported. Those participants who were unable to report for either CTB or PSG were not included in the analysis as shown in **Fig 2**. The demographic profile of participants is provided in **Supplementary Table 1**. In four participants, sleep diary data for some days was missing in either the baseline or post-intervention data but was < 10%. This was imputed with the mean of rest data values. This was either in baseline (n=3) or in post-intervention (n=1) and none in both baseline and post-intervention. Analysis was done on these completed sleep diaries (n=38). Sleep diary analysis is shown in **Table 1**. We found a significant improvement in total sleep time, sleep quality, sleep onset latency, and WASO which was reported subjectively by the patients using sleep diary.

Fig 2: Details of the Participants Screened and Finally Analyzed

Table 1: Sleep Diary Parameters of Participants Before (baseline) and After Four Weeks of Yoga-Nidra Practice (post intervention) After Training:

**Sr. No.**	**Sleep diary parameter**	**Baseline-BL Mean (SD)**	**Post-Intervention-PI Mean (SD)**	**Wilcoxon Test (P-value)**	**Difference: PI-BL Mean (SD)**	**95% CI (PI-BL)**	**%Change = (PI-BL)/BL*100**
1	sdTIB(min)	409·391 (80·277)	427·514 (66·959)	<0·0001	18·122 (98·999)	(9·691, 26·554)	4·427%
2	sdTST(min)	383·949	408·963	<0·0001	25·013	(16·435,	6·515%

		(80·267)	(69·595)		(100·720)	33·592)	
3	sdSOL(min)	21·754 (21·052)	16·309 (17·902)	<0·0001	-5·445 (25·319)	(-7·601, -3·289)	-25·030%
4	sdWASO(min)	3·991 (9·929)	2·291 (5·717)	<0·0001	-1·700 (10·699)	(-2·612, -0·789)	42·596%
5	sdSQ	7·943 (1·299)	8·247 (1·164)	<0·0001	0·305 (1·492)	(0·178, 0·432)	3·840%
6	sdSE(%)	93·751 (5·608)	95·569 (4·629)	<0·0001	1·819 (6·704)	(1·248, 2·390)	1·940%

Notes- sdTIB(min): Time in bed in minutes, sdTST(min): Total sleep time in minutes, sdSOL(min): Sleep onset latency in minutes, sdWASO(min): Wake after sleep onset in minutes, sdSQ: Sleep quality (subjective rating between 0-10), sdSE(%): Sleep efficiency calculated as (TIB/TST)*100

Polysomnography data (baseline; post-intervention; p-value) of TIB (428·09±82·13; 423·02±72·96; 0·676), TST (372·36±68·46; 387·70±74·94; 0·241), and of Total Non-REM (340·73±111·95; 341·88±73·48; 0·953) was not significant. Pre-post-intervention analysis of other PSG parameters is shown in **Fig 3**. Our results showed an objective improvement in nighttime sleep after yoga nidra practice in novices. Objectively with PSG, we found an increase in sleep efficiency, improvement in WASO and a significant improvement in delta sleep (%) of slow wave sleep. The mechanism of improvement of slow wave sleep at night time sleep when yoga nidra was done during the morning hours probably can be explained due to the reduction in the sympathetic drive and increase in parasympathetic drive (29). However, the exact mechanism is still not elucidated. The objective improvement of the delta sleep in slow wave sleep is an important factor in improvement of sleep quality. Enhancement of slow wave sleep is reported using auditory stimuli during sleep, gaboxadol, tiagabine and transcranial direct cranial stimulation(30–32). Slow wave sleep enhancement has been found to improve attention, learning, memory and performance(31–33). Slow waves during sleep have been directly or indirectly linked to synaptic strength as a part of synaptic homeostasis theory (34).

Fig 3: Comparison of Objective Sleep Parameters using Polysomnography in Subjects Before (baseline) and After (post intervention) Yoga Nidra Practice. (Note- $: p-values computed using Wilcoxon signed rank test. Rest all p-values are computed using two-tailed paired t-test.)

Cognition testing battery results for test condition wise and testing time wise comparisons in reaction time are shown in **Fig 4** and **Fig 5** respectively. The comparisons of condition wise and testing time in accuracy scores are shown in **Supplementary Figure 1** and **Supplementary Figure 2** respectively. Master sheet of CTB results in Mean (SD) is given in **Supplementary Table 2**. BART results for ‘adjusted number of pumps’, ‘risk propensity’, ‘cash collected’, and ‘number of balloons burst’ are shown in **Supplementary Figure 3 and Supplementary Figure 4** for test condition and testing time wise comparisons respectively. PVT result of ‘number of false starts’ is shown in **Supplementary Figure 5**.

Fig 4: Cognitive Testing Battery Results Showing Reaction Time (Mean (SD)) in (**A**)MPT, (**B)**VOLT, (**C**)NBACK, (**D**)AIM, (**E**)LOT, (**F**)ERT, (**G**)MRT, (**H**)DSST, (**I**)BART, and (**J**)PVT with Test Condition Wise Comparison. (Comparison model for each test condition i.e. CEC, CEO, YN1W, and YN2W is shown below each time i.e. Mr1, Mr2, and PL. Significant post-hoc p-values are also depicted)
#: One-Way Repeated Measure (RM) Analysis of Variance (ANOVA) p-values

$: Post-hoc analysis (pairwise comparison) is done using paired t-test with Bonferroni correction (significant changes indicated by p-values given above the bars).
@: Friedman's test p-value (if data is not normally distributed) &: Post-hoc analysis (pairwise comparison) is done using Wilcoxon Signed-Rank test with Bonferroni correction (significant changes indicated by p-values given above the bars).
Mr1: Test just before CEC, CEO, YN1W or YN2W ; Mr2: Tests conducted just after CEO, C EC, YN1W or YN2W; PL: Test conducted after the lunch

Fig 5: Cognitive Testing Battery Results Showing Reaction Time (Mean (SD)) in (**A**)MPT, (**B**)VOLT, (**C**)NBACK, (**D**)AIM, (**E**)LOT, (**F**)ERT, (**G**)MRT, (**H**)DSST, (**I**)BART, and (**J**)PVT with Time Wise Comparison. (Comparison model for each test condition i.e. CEC, CEO, YN1W, and YN2W is shown for each time i.e. Mr1, Mr2, and PL. Significant post-hoc p-values are also depicted)
#: One-Way Repeated Measure (RM) Analysis of Variance (ANOVA) p-values
$: Post-hoc analysis (pairwise comparison) is done using paired t-test with Bonferroni correction (significant changes indicated by p-values given above the bars).
@: Friedman's test p-value (if data is not normally distributed) &: Post-hoc analysis (pairwise comparison) is done using Wilcoxon Signed-Rank test with Bonferroni correction (significant changes indicated by p-values given above the bars).
Mr1: Test just before CEC, CEO, YN1W or YN2W ; Mr2: Tests conducted just after CEO, C EC, YN1W or YN2W; PL: Test conducted after the lunch

Yoga nidra practice showed significant improvement in reaction time with no deterioration in accuracy of all cognitive battery tests i.e., MPT, VOLT, NBACK, AIM, LOT, ERT, MRT, DSST, BART, and PVT. This implies an increase in processing speed. Certain tests like AIM, ERT, NBACK, and VOLT actually showed an increase in accuracy too. This is possible due to the effects of the practice either directly or indirectly on the various brain regions involved in performing these tests. It is possible that these effects are due to the local sleep seen in central, occipital and parietal areas during the rotation of consciousness in yoga nidra practice as brought out by Datta et al in a previous study on novices(18). Local sleep has been found to enhance task performance (35). Though not studied in this study, but probably the increase in synaptic strength as per the synaptic hypothesis might also be responsible for these findings(34).

Accuracy improved in Abstract Matching test which is specifically related to prefrontal cortex and includes abstraction and concept formation. This accuracy was found to be increased in early morning which may be as a direct or indirect effect related to improved sleep after yoga nidra practice. Slow wave sleep is essential for enhanced prefrontal cortex activity. Working memory as shown with NBACK test also showed significant improvement with yoga nidra practice. The actual number of 'adjusted number of pumps' on balloons collected as cash, showed a significant increase in post lunch session after two weeks of practice. This meant that the number of bursts on the balloons only which were encashed was higher. ERT showed a significant deterioration in the early morning task scores, though, immediately after yoga nidra practice, it did not show any significant change in scores as compared to baseline. This was further studied by analyzing the type of stimuli where there was a change. ERT analysis for various types of stimuli is shown in **Supplementary Table 3**. On further analysis, it was evident that yoga nidra intervention only showed deterioration in neutral stimuli recognition without affecting the emotion recognition of happy stimuli and in fact improved recognition of anger and fear stimuli. The exact mechanism of this cannot be understood and would require further study. The relative improvement in non-neutral emotional recognition task is also interesting and requires more deliberation with special emphasis on emotion recognition and thus future study may aim at the effects of yoga nidra practice on social interaction.

12 participants volunteered for daytime EEG recording during CTB. To understand the mechanism of the effect of the yoga nidra practice during these cognitive tasks EEG PSD values at O1, F3, and C3 were analyzed. Summary of significant test results in EEG analysis of power spectra density using Python are shown in **Table 2** and details are given in **Supplementary Table 4**. These showed significant changes in VOLT, in which accuracy of CTB was also found to be increased. Significant increase in PSD in delta and theta frequencies was seen at O1 location after two weeks of yoga nidra practice. At C3 location also, PSD of

theta remained significantly increased, immediately after yoga nidra practice during VOLT. A local sleep type phenomenon with some direct or indirect effects on areas associated with VOLT i.e. middle-temporal cortex and hippocampus, might explain this positive effect on spatial learning in memory. In our study, it is also interesting to note that after the practice also, delta propensity continued without reduction in accuracy, rather improvement in reaction time. It can only be postulated that increased PSD of delta waves implying enhancement of slow waves may be due to the increased strength of synapses(36) as highlighted by Tononi et al. Increased delta propensity during tasks on EEG PSD values, especially in the early morning readings after two weeks of yoga nidra practice, hints also at a not so immediate effect of practice, and might be a post good night sleep effect after practicing yoga nidra for two weeks in the mornings.

Table 2: EEG PSD Significant Results in Frequency Bands during CTB at Various EEG Locations

Sr. No.	CBT	EEG Channel	Test Time	Frequency Band	Test Condition: Mean (SD)	Post-hoc Test (P-value)	Difference*	95% CI for Difference*	% Change
1	VOLT	O1	Mr1	delta/alpha1	CEC: 5·795(4·573)<br>CEO: 2·548(1·079)	0·027	-3·250	(-6·21,-0·286)	-56·083%
				delta/alpha2	CEC: 6·547(5·049)<br>CEO: 2·774(1·146)	0·026	-3·770	(-7·19, -0·356)	-57·584%
				delta/theta2	CEC: 4·202(2·936)<br>CEO: 2·141(0·779)	0·031	-2·060	(-3·970, -0·148)	-49·024%
					CEC: 4·202(2·936)<br>YN2W: 2·084(0·503)	0·043	-2·120	(-4·180, -0·052)	-50·452%
				Theta1(4-6Hz)	CEO: 0·118(0·024)<br>YN2W: 0·157(0·033)	0·014	0·039	(0·006, 0·072)	33·051%
					YN1W: 0·113(0·021)<br>YN2W: 0·157(0·033)	0·014	0·045	(0·007, 0·082)	39·823%
				Theta2(6-8Hz)	CEC: 0·069(0·026)<br>YN2W: 0·107(0·023)	0·025	0·038	(0·004, 0·073)	55·072%
					YN1W: 0·074(0·019)<br>YN2W: 0·107(0·023)	0·049	0·033	(0·001, 0·066)	44·595%
				theta2/alpha2	CEO: 1·329(0·437)<br>YN2W: 2·056(0·796)	0·024	0·728	(0·078, 1·380)	54·778%
			PL	Theta2(6-8Hz)	CEO: 0·075(0·017)<br>YN2W: 0·102(0·016)	0·021	0·028	(0·003, 0·052)	37·333%
		C3	Mr2	Theta2(6-8Hz)	ECE: 0·055(0·019)<br>YN1W: 0·095(0·041)	0·041	0·040	(0·001, 0·080)	72·727%
2	BART	O1	Mr2	delta/beta	CEC: 0·951(0·458)<br>YN2W: 2·058(1·275)	0·040$	1·110	(0·039,2·180)	116·719%
					CEO: 0·946(0·496)<br>YN2W: 2·058(1·275)	0·029$	1·110	(0·090, 2·130)	117·336%
3	PVT	O1	Mr2	Theta2(6-8Hz)	CEO: 0·071(0·017)<br>YN1W: 0·098(0·028)	0·040	0·027	(0·001, 0·053)	38·028%
4	ERT	F3	PL	delta/alpha1	CEC: 4·506(1·483)<br>YN1W: 10·257(5·90)	0·026	5·750	(0·570, 10·90)	127·608%
				delta/alpha2	CEC: 6·413(2·641)<br>YN1W: 15·347(10·086)	0·036	8·930	(0·481, 17·400)	139·248%
					CEO: 6·613(3·535)<br>YN1W: 15·347(10·086)	0·034	8·730	(0·529, 16·900)	132·013%
				delta/theta1	CEC: 1·737(0·224)<br>YN1W: 2·224(0·387)	0·029	0·487	(0·042, 0·932)	28·037%
				delta/theta2	CEC: 3·078(0·644)<br>YN1W: 5·345(2·179)	0·031	2·270	(0·168, 4·370)	73·749%
				Theta1/alpha2	CEO: 3·440(1·423)<br>YN1W: 6·753(3·806)	0·042	3·310	(0·091, 6·530)	96·221%
5	LOT	F3	PL	delta/theta1	CEC: 1·819(0·460)<br>YN1W: 2·348(0·231)	0·048	0·530	(0·004, 1·050)	29·137%
6	MPT	C3	Mr2	Theta1(4-6Hz)	CEC: 0·065(0·029)<br>YN1W: 0·114(0·021)	0·024	0·049	(0·005, 0·092)	75·385%
7	MRT	C3	Mr1	delta/beta	CEC: 1·669(0·372)<br>CEO: 0·932(0·424)	0·048$	-0·737	(-1·29, -0·184)	-44·158%

Note:
One-way ANOVA model was used for checking significant change and post-hoc analysis is done using Tukey post hoc test except at $ where Kruskal Walli's test was used and post-hoc is done using Wilcoxon test·

VOLT: Visual Object Learning Task, BART: Balloon Analog Risk Task, PVT: Psychomotor Vigilance Task, ERT: Emotion Recognition Task, LOT: Line Orientation Task, MPT: Motor Praxis Task, MRT: Matrix Reasoning Task

CEO: Control with Eyes Open, CEC: Control with Eyes Close, YN1W: At the end of one week of Yoga-nidra practice after training, YN2W: At the end of two weeks of Yoga-nidra practice after training·
Mr1: Test just before CEC, CEO, YN1W or YN2W, Mr2: Tests conducted just after CEO, CEC, YN1W or YN2W, PL: Tests conducted after the lunch.
*Difference is calculated as difference between two PSD parameters of two test conditions given in 'Test Condition' column.

The study highlights the important effect of yoga nidra practice on the cognitive processing. Though there are limitations of this study as it demonstrates effects of yoga nidra practice for only two weeks and the EEG locations studied while doing the cognitive tasks were also limited, results with larger samples for longer durations may give a better perspective. This study opens up an opportunity for the use of an easy-to-do practice of yoga nidra for population health using standardized supervised model(6,12). The study highlights the possible role of this practice in improving sleep and in promoting learning and memory amongst healthy participants. It might hold promise for patients with mild learning disability and mild cognition deterioration specially in aging(37). In the post-pandemic times, sleep is commonly found to be affected, which creates a risk of multiple disorders including neuropsychiatric disorders(38). Planning large population based studies of different cultures(5) may also help assess its effects on insomnia globally. Awareness of physicians at the primary care level about this simple to do practice, bringing out clear cut guidelines and sleep management by healthcare workers and primary care physicians in general thus may be vital to ensure wellbeing of the society as it emerges from the effects of pandemic (39).

Acknowledgement:

Authors acknowledge Department of Science and Technology, Government of India for financial support vide Reference No. DST Satyam 2018/457 dated 28 May 2020 under Science and Technology for Yoga and Meditation (SATYAM) to carry out this work. Authors thank Dr CV Apte for reviewing the manuscript and thank the participants and staff for the support in conducting the study.

Funding Statement:

This work was supported by Department of Science and Technology under Science and Technology for Yoga and Meditation (SATYAM), India, received vide their sanction order DST Satyam 2018/457 dated 28 May 2020.

Supplementary Information

Supplementary Figure 1: Cognitive Testing Battery Results Showing Accuracy Measure (Mean(SD)) in (**A**)MPT, (**B**)VOLT, (**C**)NBACK, (**D**)AIM, (**E**)LOT, (**F**)ERT, (**G**)MRT, (**H**)DSST, and (**I**)PVT with Test Condition Wise Comparison. (Comparison model for each test condition i.e. CEC, CEO, YN1W, and YN2W is shown below each time i.e. Mr1, Mr2, and PL. Significant post-hoc p-values are also in are also depicted)

#: One-Way Repeated Measure (RM) Analysis of Variance (ANOVA) p-values
$: Post-hoc analysis (pairwise comparison) is done using paired t-test with Bonferroni correction (significant changes indicated by p-values given above the bars).
@: Friedman's test p-value (if data is not normally distributed)
&: Post-hoc analysis (pairwise comparison) is done using Wilcoxon Signed-Rank test with Bonferroni correction (significant changes indicated by p-values given above the bars).

Mr1: Test just before CEC, CEO, YN1W or YN2W	Mr2: Tests conducted just after CEO, C EC, YN1W or YN2W
PL: Test conducted after the lunch	CEO: Control with Eyes Open
CEC: Control with Eyes Close	YN1W: At the end of one week of Yoga-Nidra practice after training
YN2W: At the end of two weeks of Yoga-Nidra practice after training

Supplementary Figure 2: Cognitive Testing Battery Results Showing Accuracy Measure (Mean(SD)) in (**A**)MPT, (**B**)VOLT, (**C**)NBACK, (**D**)AIM, (**E**)LOT, (**F**)ERT, (**G**)MRT, (**H**)DSST, and (**I**)PVT with Time Wise Comparison. (Comparison model for each test condition i.e. CEC, CEO, YN1W, and YN2W is shown below each time i.e. Mr1, Mr2, and PL. Significant post-hoc p-values are also in are also depicted)

#: One-Way Repeated Measure (RM) Analysis of Variance (ANOVA) p-values
$: Post-hoc analysis (pairwise comparison) is done using paired t-test with Bonferroni correction (significant changes indicated by p-values given above the bars).
@: Friedman's test p-value (if data is not normally distributed)
&: Post-hoc analysis (pairwise comparison) is done using Wilcoxon Signed-Rank test with Bonferroni correction (significant changes indicated by p-values given above the bars).

Mr1: Test just before CEC, CEO, YN1W or YN2W	Mr2: Tests conducted just after CEO, C EC, YN1W or YN2W
PL: Test conducted after the lunch	CEO: Control with Eyes Open
CEC: Control with Eyes Close	YN1W: At the end of one week of Yoga-Nidra practice after training
YN2W: At the end of two weeks of Yoga-Nidra practice after training

Supplementary Figure 3: BART results (Mean(SD)) of **A**) Adjusted Number of Pumps, **B**)Total cash collected($), **C**) Risk Propensity, and **D**) Number of Balloons Burst Before (baseline) and After (post-intervention) Yoga Nidra Practice Test Condition Wise. (P-values for time wise and test condition wise comparison model is shown below i.e. for Mr1, Mr2, and PL; and CEC, CEO, YN1W, and YN2W respectively. Significant post-hoc p-values are also depicted)

Notes- #: One-Way Repeated Measure (RM) Analysis of Variance (ANOVA) p-values, $: Post-hoc analysis (pairwise comparison) is done using paired t-test with Bonferroni correction (significant changes indicated by p-values given above the bars). , @: Friedman's test p-value (if data is not normally distributed), &: Post-hoc analysis (pairwise comparison) is done using Wilcoxon Signed-Rank test with Bonferroni correction (significant changes indicated by p-values given above the bars).
Mr1: Test just before CEC, CEO, YN1W or YN2W	Mr2: Tests conducted just after CEO, C EC, YN1W or YN2W
PL: Test conducted after the lunch	CEO: Control with Eyes Open
CEC: Control with Eyes Close	YN1W: At the end of one week of Yoga-Nidra practice after training
YN2W: At the end of two weeks of Yoga-Nidra practice after training

Supplementary Figure 4: BART results (Mean (SD)) of **A**) Adjusted Number of Pumps, **B**)Total cash collected($), **C**) Risk Propensity, and **D**) Number of Balloons Burst Time Wise Before (baseline) and After (post-intervention) Yoga Nidra Practice. (P-values for time wise and test condition wise comparison model is shown below i.e., for Mr1, Mr2, and PL; and CEC, CEO, YN1W, and YN2W respectively. Significant post-hoc p-values are also depicted)

Notes- #: One-Way Repeated Measure (RM) Analysis of Variance (ANOVA) p-values, $: Post-hoc analysis (pairwise comparison) is done using paired t-test with Bonferroni correction (significant changes indicated by p-values given above the bars). , @: Friedman's test p-value (if data is not normally distributed), &: Post-hoc analysis (pairwise comparison) is done using Wilcoxon Signed-Rank test with Bonferroni correction (significant changes indicated by p-values given above the bars).
Mr1: Test just before CEC, CEO, YN1W or YN2W	Mr2: Tests conducted just after CEO, C EC, YN1W or YN2W
PL: Test conducted after the lunch	CEO: Control with Eyes Open
CEC: Control with Eyes Close	YN1W: At the end of one week of Yoga-Nidra practice after training
YN2W: At the end of two weeks of Yoga-Nidra practice after training

Supplementary Figure 5: PVT-Number of False Starts (Mean (SD)) of Participants Before (baseline) and After (post-intervention) Yoga Nidra Practice Comparison in Test Condition wise (6(**A**)) and Time wise (6(**B**)).Comparison model for each test condition i.e. CEC, CEO, YN1W, and YN2W is shown below each time i.e. Mr1, Mr2, and PL. Significant post-hoc p-values are also depicted.

Supplementary Table 1: Demographic profile of participants

Supplementary Table 2: Cognitive Testing Battery (CTB) parameters (Mean (SD)) of all tests in the participants.

Supplementary Table 3: Emotion Reaction Time (ERT) Emotion-wise Analysis of Accuracy

Supplementary Table 4: Significant EEG PSD results in Frequency Bands during CTB at Various EEG Locations

References:

1. Woodyard C. Exploring the therapeutic effects of yoga and its ability to increase quality of life. Int J Yoga. 2011 Jul;4(2):49–54.
2. Gothe NP, McAuley E. Yoga and Cognition: A Meta-Analysis of Chronic and Acute Effects. Psychosomatic Medicine. 2015 Sep;77(7):784–97.
3. Jeter PE, Slutsky J, Singh N, Khalsa SBS. Yoga as a Therapeutic Intervention: A Bibliometric Analysis of Published Research Studies from 1967 to 2013. The Journal of Alternative and Complementary Medicine. 2015 Oct;21(10):586–92.
4. Satyananda Saraswati, Bihar School of Yoga. Yoga nidra. Munger: Yoga Publications Trust; 1998.
5. Hoye S, Reddy S. Yoga-nidra and hypnosis. International Journal of Health Promotion and Education. 2016 May 3;54(3):117–25.
6. Datta K, Tripathi M, Verma M, Masiwal D, Mallick HN. Yoga nidra practice shows improvement in sleep in patients with chronic insomnia: A randomized controlled trial. NMJI. 2021 Oct 22;34(3):143–50.
7. Datta K, Yadav N, Narawade Y, Mahajan U, Joseph T, Kumar A. Sleep Strategies for Sportsmen; What can they be? A Randomised Controlled Trial. Sleep Vigilance [Internet]. 2022 Jan 4 [cited 2022 Feb 18]; Available from: https://link.springer.com/10.1007/s41782-021-00188-8
8. Rani K, Tiwari S, Singh U, Agrawal G, Ghildiyal A, Srivastava N. Impact of Yoga Nidra on psychological general wellbeing in patients with menstrual irregularities: A randomized controlled trial. Int J Yoga. 2011 Jan;4(1):20–5.
9. Rani K, Tiwari S, Singh U, Singh I, Srivastava N. Yoga Nidra as a complementary treatment of anxiety and depressive symptoms in patients with menstrual disorder. Int J Yoga. 2012 Jan;5(1):52–6.
10. Stankovic L. Transforming trauma: a qualitative feasibility study of integrative restoration (iRest) yoga Nidra on combat-related post-traumatic stress disorder. Int J Yoga Therap. 2011;(21):23–37.
11. Pritchard M, Elison-Bowers P, Birdsall B. Impact of integrative restoration (iRest) meditation on perceived stress levels in multiple sclerosis and cancer outpatients. Stress and Health. 2009 Oct 13;26(3):233–7.
12. Datta K, Tripathi M, Mallick HN. Yoga Nidra: An innovative approach for management of chronic insomnia- A case report. Sleep Science Practice. 2017 Dec;1(1):7.

13. Alhola P, Polo-Kantola P. Sleep deprivation: Impact on cognitive performance. Neuropsychiatr Dis Treat. 2007 Oct;3(5):553–67.

14. Zaccaro A, Riehl A, Piarulli A, Alfì G, Neri B, Menicucci D, et al. The Consciousness State of Traditional Nidrâ Yoga/Modern Yoga Nidra: Phenomenological Characterization and Preliminary Insights from an EEG Study. International Journal of Yoga Therapy. 2021 Jan 1;31(1):Article_14.

15. Kjaer TW, Bertelsen C, Piccini P, Brooks D, Alving J, Lou HC. Increased dopamine tone during meditation-induced change of consciousness. Brain Res Cogn Brain Res. 2002 Apr;13(2):255–9.

16. Lou HC, Kjaer TW, Friberg L, Wildschiodtz G, Holm S, Nowak M. A 15O-H2O PET study of meditation and the resting state of normal consciousness. Hum Brain Mapp. 1999;7(2):98–105.

17. Braboszcz C, Cahn BR, Levy J, Fernandez M, Delorme A. Increased Gamma Brainwave Amplitude Compared to Control in Three Different Meditation Traditions. Najbauer J, editor. PLoS ONE. 2017 Jan 24;12(1):e0170647.

18. Datta K, Mallick HN, Tripathi M, Ahuja N, Deepak KK. Electrophysiological Evidence of Local Sleep During Yoga Nidra Practice. Front Neurol. 2022;13:910794.

19. Moszeik EN, von Oertzen T, Renner KH. Effectiveness of a short Yoga Nidra meditation on stress, sleep, and well-being in a large and diverse sample. Current Psychology. 2020;1–15.

20. Basner M, Savitt A, Moore TM, Port AM, McGuire S, Ecker AJ, et al. Development and Validation of the <I>Cognition</I> Test Battery for Spaceflight. Aerospace Medicine and Human Performance. 2015 Nov 1;86(11):942–52.

21. Lejuez CW, Read JP, Kahler CW, Richards JB, Ramsey SE, Stuart GL, et al. Evaluation of a behavioral measure of risk taking: The Balloon Analogue Risk Task (BART). Journal of Experimental Psychology: Applied. 2002;8(2):75–84.

22. Gramfort A, Luessi M, Larson E, Engemann DA, Strohmeier D, Brodbeck C, et al. MNE software for processing MEG and EEG data. NeuroImage. 2014 Feb;86:446–60.

23. Al-Fahoum AS, Al-Fraihat AA. Methods of EEG signal features extraction using linear analysis in frequency and time-frequency domains. ISRN Neurosci. 2014;2014:730218.

24. Alam MN, Ibrahimy MI, Motakabber SMA. Feature Extraction of EEG Signal by Power Spectral Density for Motor Imagery Based BCI. In: 2021 8th International Conference on Computer and Communication Engineering (ICCCE) [Internet]. Kuala Lumpur, Malaysia: IEEE; 2021 [cited 2022 Sep 29]. p. 234–7. Available from: https://ieeexplore.ieee.org/document/9467141/

25. Jiang X, Bian GB, Tian Z. Removal of Artifacts from EEG Signals: A Review. Sensors (Basel). 2019 Feb 26;19(5):E987.

26. Tzallas AT, Tsipouras MG, Fotiadis DI. Automatic seizure detection based on time-frequency analysis and artificial neural networks. Comput Intell Neurosci. 2007;80510.

27. Hillard B. Analysis of EEG rhythms using custom-made MatLab application for processing of data collected during neurofeedback training in ADHD subjects. [Internet]. University of Louisville; 2012 [cited 2022 Sep 29]. Available from: http://ir.library.louisville.edu/etd/620

28. Berry RB, Brooks R, Gamaldo CE, Harding SM, Lloyd RM, Marcus CL and Vaughn BV for the American Academy of Sleep Medicine. The AASM Manual for the Scoring of Sleep and Associated Events: Rules, Terminology and Technical Specifications, Version 2.0.3. www.aasmnet.org, Darien, Illinois: American Academy of Sleep Medicine, 2014.

29. Patra S, Telles S. Heart rate variability during sleep following the practice of cyclic meditation and supine rest. Appl Psychophysiol Biofeedback. 2010 Jun;35(2):135–40.

30. Lang N, Siebner HR, Ward NS, Lee L, Nitsche MA, Paulus W, et al. How does transcranial DC stimulation of the primary motor cortex alter regional neuronal activity in the human brain?: tDCS-induced changes of rCBF. European Journal of Neuroscience. 2005 Jul 21;22(2):495–504.

31. Walsh JK, Snyder E, Hall J, Randazzo AC, Griffin K, Groeger J, et al. Slow Wave Sleep Enhancement with Gaboxadol Reduces Daytime Sleepiness During Sleep Restriction. Sleep. 2008 May;31(5):659–72.

32. Walsh J, Zammit G, Schweitzer P, Ondrasik J, Roth T. Tiagabine enhances slow wave sleep and sleep maintenance in primary insomnia. Sleep Medicine. 2006 Mar;7(2):155–61.

33. Datta K, Kumar A, Guru C. Enhancement of performance in an elite archer after non-pharmacological intervention to improve sleep. Medical Journal Armed Forces India. 2018 Oct 1;

34. Tononi G, Cirelli C. Sleep function and synaptic homeostasis. Sleep Medicine Reviews. 2006 Feb;10(1):49–62.

35. Huber R, Ghilardi M, Massimini M, Tononi G. Local sleep and learning. Nature. 2004 Jul 1;430:78–81.

36. Tononi, G., Riedner, B. A., Hulse, B. K., Ferrarelli, F., and Sarasso, S. Enhancing sleep slow waves with natural stimuli. Medicamundi. 2010; 54: 73–79.

37. Grimaldi D, Papalambros NA, Zee PC, Malkani RG. Neurostimulation techniques to enhance sleep and improve cognition in aging. Neurobiology of Disease. 2020 Jul;141:104865.

38. Datta K. Biological Rhythm and Neuropsychiatric Disorders. In: Gupta R, Neubauer DN, Pandi-Perumal SR, editors. Sleep and Neuropsychiatric Disorders [Internet]. Singapore: Springer Nature Singapore; 2022 [cited 2022 May 18]. p. 53–63. Available from: https://link.springer.com/10.1007/978-981-16-0123-1_3

39. Datta K, Shrivastava D. Making Sense of Sleep Medicine: A Hands-On Guide [Internet]. 1st ed. Boca Raton: CRC Press; 2022 [cited 2022 Dec 10]. Available from: https://www.taylorfrancis.com/books/9781003093381

Click here to access/download;Figure;Fig1.tif

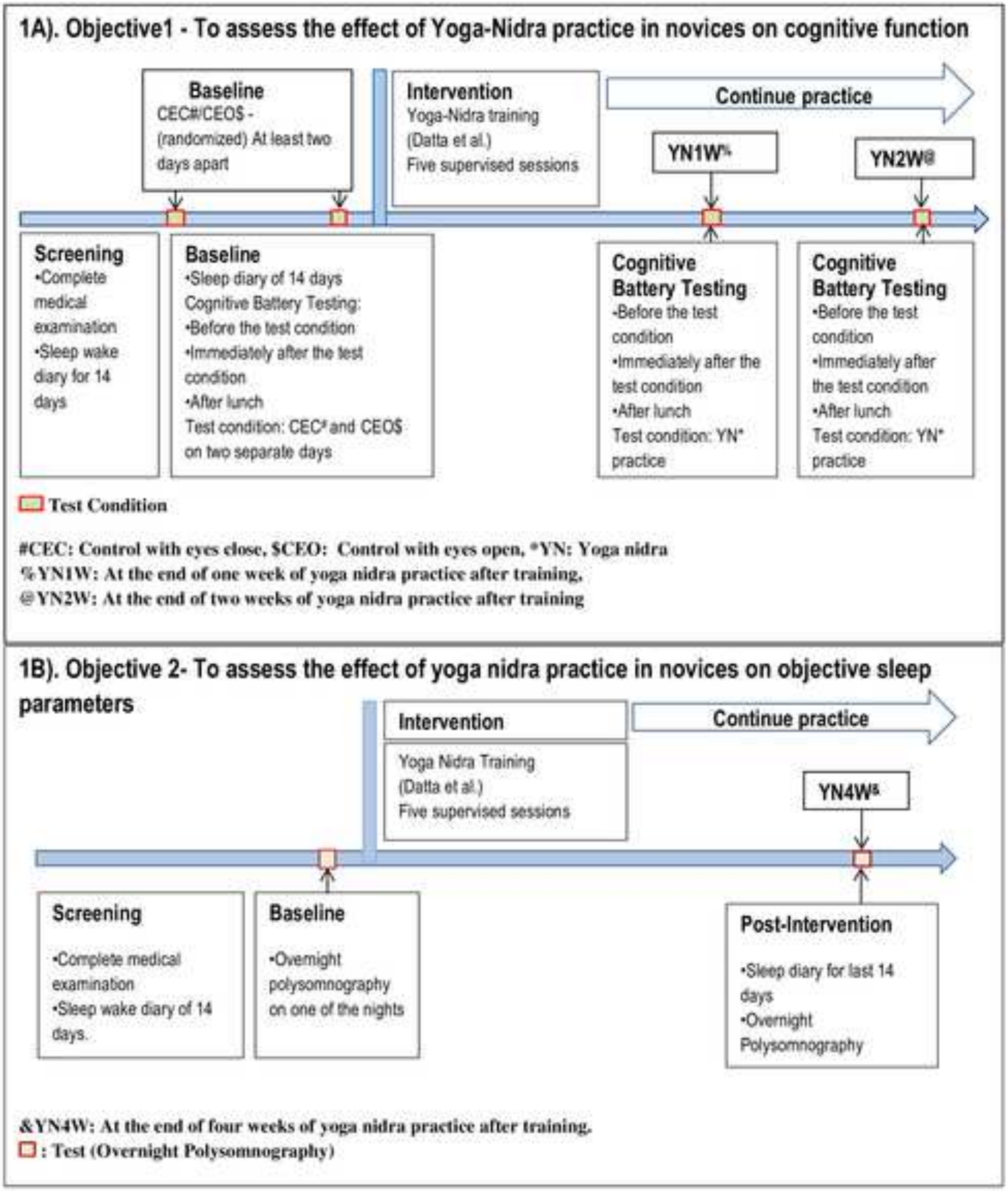

Click here to access/download;Figure;Fig 2.TIF

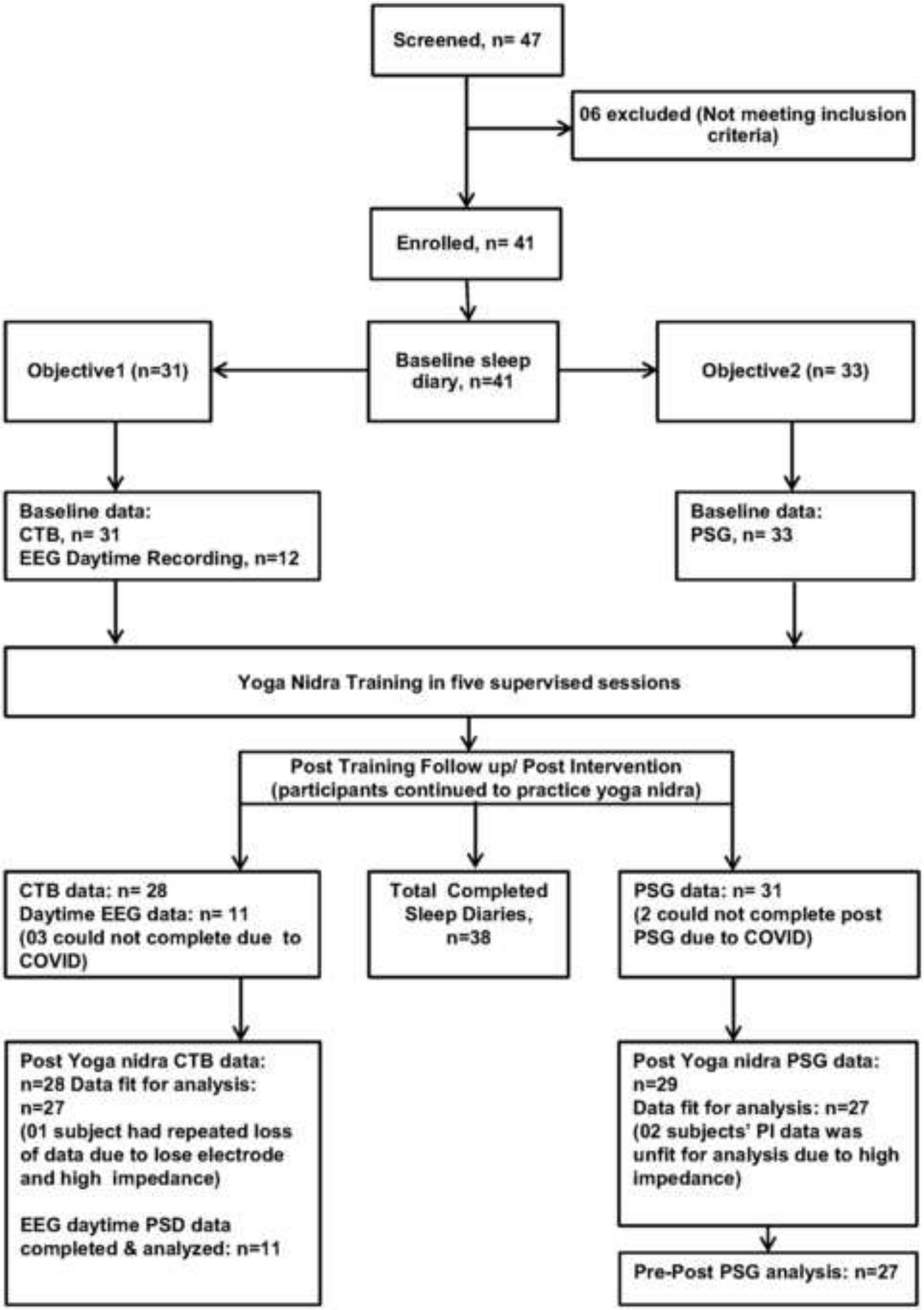

Click here to access/download;Figure;Fig3.tif

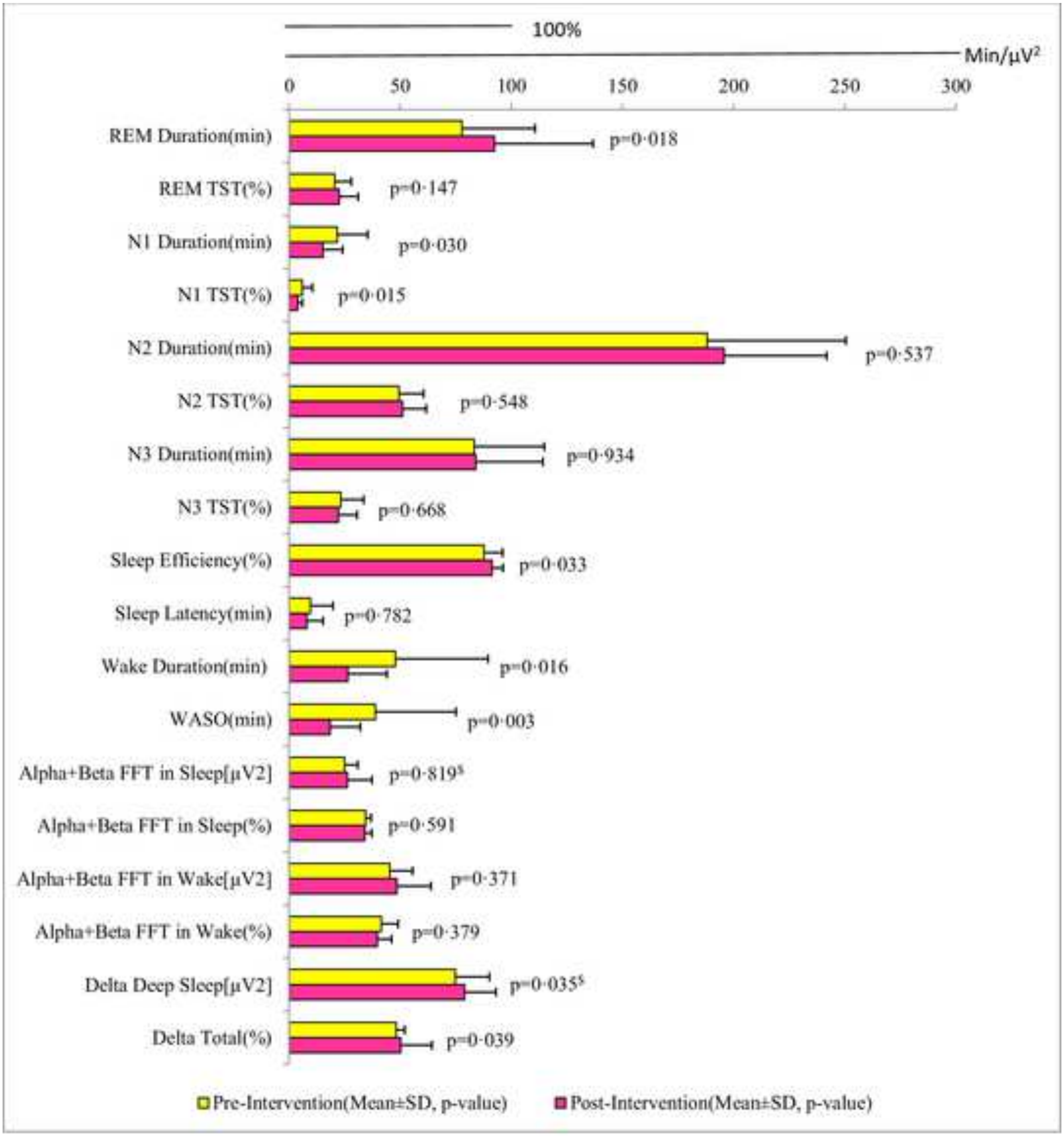

$: p-values computed using Wilcoxon signed rank test and rest all p-values are computed using two-tailed paired t-test.

Figure 3: PSG Objective Sleep Parameters Comparison in Subjects before (baseline) and after (post-intervention) yoga nidra training

Click here to access/download;Figure;Fig4.tif

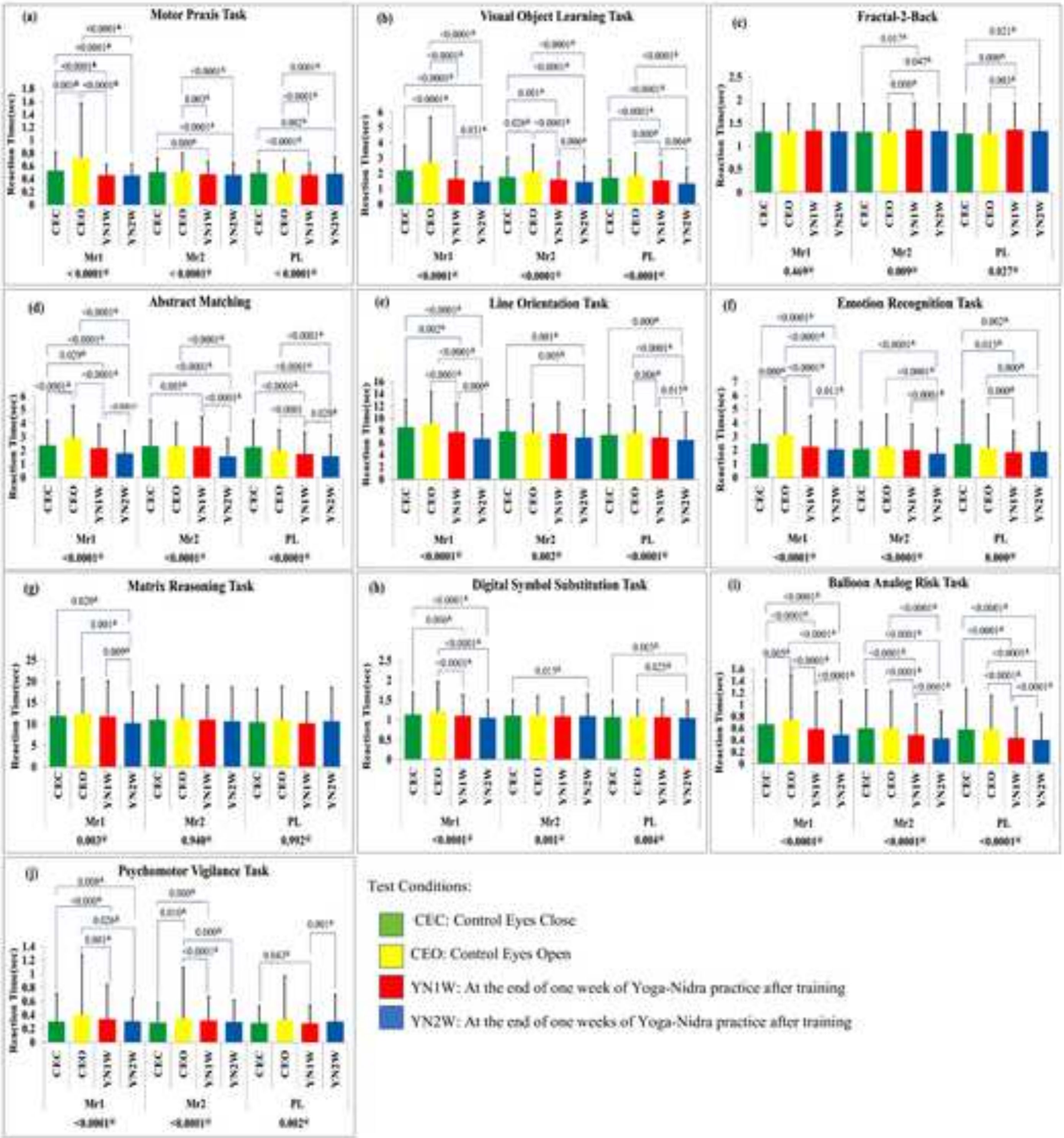

Click here to access/download;Figure;Fig5.tif

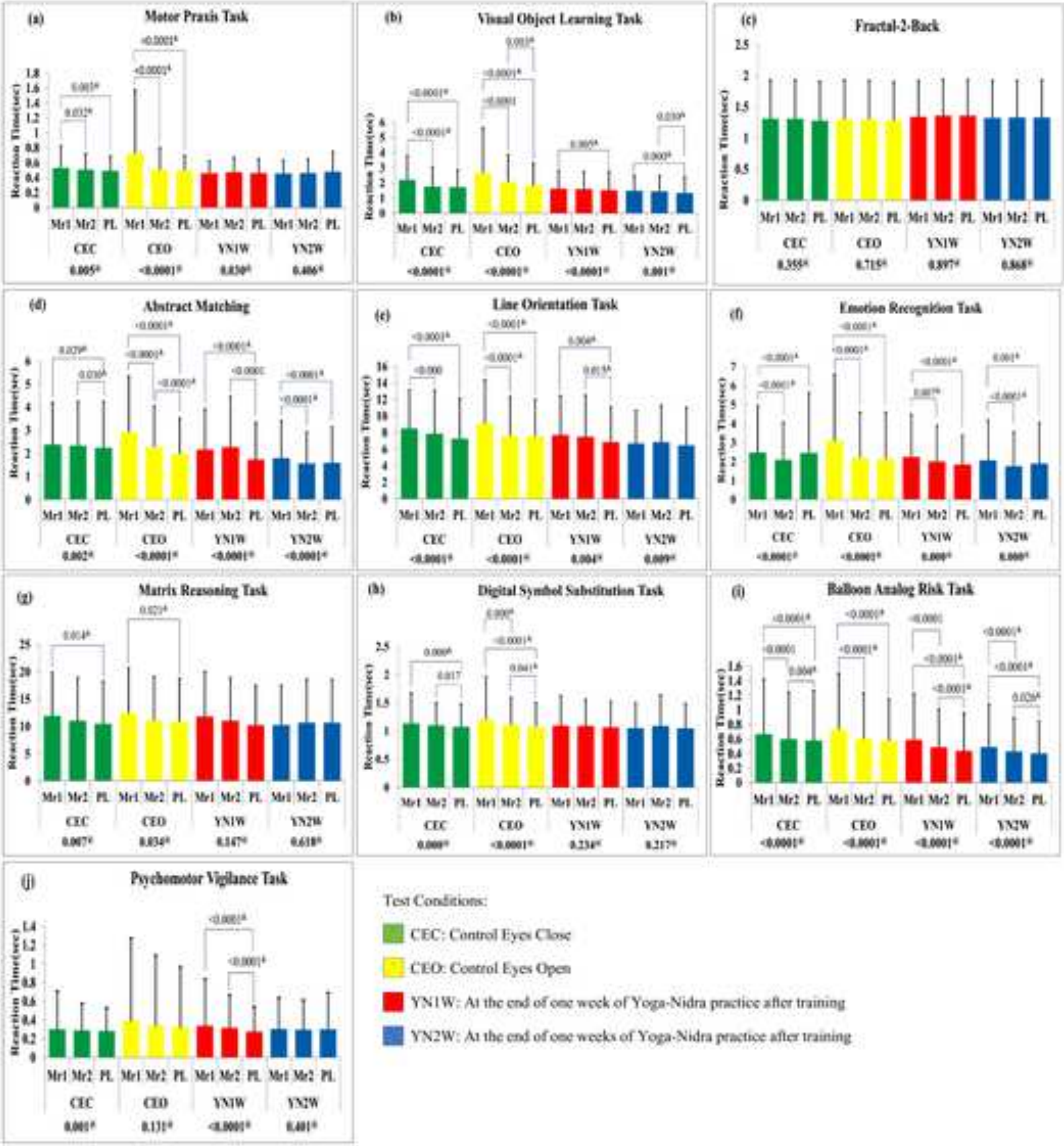

Click here to access/download
Supporting Information
Supplementary Figure 1.JPEG

Click here to access/download
Supporting Information
Supplementary Figure 2.JPEG

Click here to access/download
Supporting Information
Supplementary Figure 3.JPEG

Click here to access/download
Supporting Information
Supplementary Figure 4.JPEG

Click here to access/download
Supporting Information
Supplementary Figure 5.JPEG

Click here to access/download
Supporting Information
Supplementary Table 1.XLSX

Click here to access/download
Supporting Information
Supplementary Table 2.DOCX

Click here to access/download
Supporting Information
Supplementary Table 3.DOCX

Click here to access/download
Supporting Information
Supplementary Table 4.DOCX

Preprint PDF

Click here to access/download
Preprint PDF
2023.01.27.23284927v1.full.pdf
