# Supplementary material for: Improved sleep, cognitive processing and enhanced learning and memory task accuracy with Yoga nidra practice in novices
Source: PLoS One. 2023 Dec 13;18(12):e0294678. doi: 10.1371/journal.pone.0294678 (PMC10718434; doi:10.1371/journal.pone.0294678)
Supplement: Attachment — Submitted filename: plosResponse to Reviewer.docx [file pone.0294678.s011.docx]

**PONE-D-23-21201: Response to Reviewer’s Comments**

Authors thank the reviewers for their valuable time and their comments. The point to point reply is submitted and reference to the line numbers in the manuscript with track changes is provided as a ready reference for needful please.

| Reviewer No. | Reviewer’s comments | Reply |
| --- | --- | --- |
| Reviewer #1 | Dear authors, thank you for the possibility to review this very interesting paper. I find it very relevant for publication as objective parameters of testing always expand knowledge of subjective questionnaires. Yet, there are some remarks I would find really important to add before publication as they would strengthen the scientific standards of your work:  Please report effect sizes, at abstract as well as at result’s section. | Effect sizes are added as suggested |
|  | Please discuss the very important limitation of not using an active control group as literature suggests the effects could also occur through other positive interventions. | Thank you for your comment. This is a pilot study using pre-post intervention design. A need for a randomised controlled trial has been added in the amended manuscript (from line 296 to 298). |
|  | Also please discuss thoroughly why you exclude women and state this as limitation. | Added in the amended manuscript (from line 89 to 90). |
|  | I would find it very helpful to have an extra section strengths and limitations at an extra discussion’s section and not mixed at results’ section. | The manuscript has been arranged as per the journal submission guidelines of a combined results and discussion section. This has been done so that interpretation of the results can be explained simultaneously. |
|  | Please make clear why you decide to not call it yoga nidra meditation as a lot of other literature would do so. | Added in the amended manuscript (from line 57 to 59 and 64 to 65). |
|  | There are some small spelling mistakes I have marked at the document attached. There are also some sections where there are words missing to complete a sentence or when a technical term needs to be explained for the reader's better understanding. | Amended in the manuscript. |
|  | I wouldn't use that many statistical values at abstract but rather state very clear your results and effects sizes / meaningfulness. | Added effect sizes as suggested. |
|  | Also state limitations as an outlook here. | Added in the amended manuscript (from line 295 to 298). |
|  | Please consider APA guidelines for table e.g. table 1 and use dois for your literature section whereever possible. All the best! | Tables altered as suggested. |
| Reviewer #2 | Although this manuscript reflects a great amount of work and important findings, there are a number of glaring omissions that significantly reduce my enthusiasm for this study because a reviewer is unable to adequately gauge its methodological rigor. 1. The English used is sub-standard and negatively affects readability. The manuscript needs a thorough review for grammatical issues. | Amended throughout the manuscript. |
|  | 2. The authors assume a familiarity with the yoga nidra technique and some of the methodological details of the study that appears unwarranted and makes it difficult to evaluate. For example, how is yoga nidra different from other yoga and meditative techniques? What did supervised training consist of? | Added in the amended manuscript (from line 57 to 59 and 97 to 101). |
|  | 3. No validity data is given for the cognitive and sleep outcome measures. Has research been conducted on the subtests of the CTB? Explain in more detail about the PSG and EEG measures and why they were specifically chosen to evaluate sleep in this study. | Twelve participants volunteered out of a total number of thirty one in CTB group (refer Figure 2) and also mentioned in the manuscript from line 147 to 148. |
| Reviewer #3 | The paper offers a compelling exploration of the cognitive and sleep-related consequences of Yoga Nidra practice, with the intention of addressing a knowledge gap about the measurable outcomes of this particular practice. The novelty of the study comes in its investigation of a relatively unexplored element of the effects of Yoga Nidra. The manuscript exhibits the author's comprehension of pertinent scholarly works, citing the stress-reducing advantages of Yoga Nidra and its ability to enhance sleep quality. Nevertheless, it is imperative to incorporate citations to contemporary research papers that specifically examine the cognitive impacts of Yoga Nidra. | Added in the amended manuscript (from line 71 to 72). |
|  | The study demonstrates a strong methodological framework, incorporating thorough evaluations of cognitive function and sleep patterns using a range of tasks and polysomnography techniques. The selected methodologies are in accordance with the aims of the study, and the statistical analysis provides evidence in favor of the stated outcomes. Nevertheless, it would be beneficial to improve methodological clarity by providing more comprehensive explanations of the cognition testing battery and EEG analysis methods. | Thank you for your comments. Tables now contain the data in two decimal places and Figure 3 (y-axis) has been amended to improve clarity. |
|  | The findings are presented in a manner that effectively demonstrates the observed enhancements in cognitive tasks and sleep parameters subsequent to the practice of Yoga Nidra. The conclusions reached are consistent with the findings and discussion of the study. The article demonstrates the significance it holds for future research and the integration of complementary and alternative medicine (CAM) practices into healthcare initiatives. However, further elaboration on the study's wider societal implications and potential limitations would augment the manuscript's comprehensiveness. | Last para in the amended manuscript (lines 295 to 307) have been altered to bring this out better. |
|  | The text has a generally well-structured organization, while particular portions might benefit from more refinement to enhance overall readability. The linguistic style employed is inappropriate for a scholarly readership since it effectively presents the data analysis and findings in a manner that is not easily understood. In general, the paper provides significant insights into the impact of Yoga Nidra practice on cognition and sleep, thereby enhancing the comprehension of the potential advantages of complementary and alternative medicine (CAM) techniques. In order to enhance the quality of the paper, it is suggested that a more thorough incorporation of contemporary scholarly works be undertaken, along with certain modifications to improve the clarity of the methodology and the explanation of the consequences. | Added in the amended manuscript (from line 71 to 72). |
|  | Authors are requested in their discussion to include the following references: <https://doi.org/10.3390/bioengineering10020249>, <https://cdn.techscience.cn/files/jnm/2023/TSP_JNM-5-1/TSP_JNM_37583/TSP_JNM_37583.pdf> | Thank you for sharing this work on Sci-fi films and Identification of coronary artery disease using feature selection methods. The work is innovative and educative. However since it was not directly linked to EEG or PSG, we have not included in this manuscript. |

I look forward to your comments.

Thanking you

Corresponding Author

: 
