# Supplementary material for: Improved sleep, cognitive processing and enhanced learning and memory task accuracy with Yoga nidra practice in novices
Source: PLoS One. 2023 Dec 13;18(12):e0294678. doi: 10.1371/journal.pone.0294678 (PMC10718434; doi:10.1371/journal.pone.0294678)
Supplement: Attachment — Submitted filename: plosResponse to Reviewer1 (1).docx [file pone.0294678.s012.docx]

PONE-D-23-21201

Response to Reviewer’s Comments

Authors thank the reviewers for their valuable comments. The point to point reply is submitted for needful please.

| Reviewer No. |  |  |
| --- | --- | --- |
| Reviewer #1 | Statistical abbreviations have to be italic (APA) | Amended. |
|  | Lines 57-59, this is not precise, one part of yn is pratyahara but not all of it, and there are studies that highlight alpha waves! | Modified and clarified. |
|  | Line 68/69, there is the same source two times, online first and print versions are the same. please double check your literature if there are more doubles like this | Thank you for pointing out this error. The discrepancy has been amended and checked. |
|  | Line 70, there are two dots. Also at 273 | Amended. |
|  | 84 ff important information has always to appear also at running text, e.g. how many males took part in this study;  89 ff how long was the yn | Volunteer Males were taken as mentioned in methods section. The number of males who got recruited and finally took part in the study has been shown in figure 2 and mentioned in the results too.  Added and amended in the manuscript  ‘Yoga nidra was practiced for 20 minutes with three minutes of instructions before and after the practice.’ |
|  | Did you perform a power analysis to find out about the sufficient sample size for your study? Please report about the power of your study, at least at discussion's section about strengths/weaknesses | The study was done as a pilot project. 41 volunteers completed the study. This being a pilot study with no active groups is highlighted now as limitation in discussion. |
|  | There is still missing a comment on the value of active control groups! | Highlighted in discussion as limitation. |
|  | Line 180 (also 248) numbers are written as numbers when above 10 (APA); how many is "some"? | Amended  ‘Some’ is ‘Three’ and has been amended now |
|  | Why do you report different effect sizes? d, r and eta - please either choose one or explain why you choose three and differentiate the thresholds of small, medium, big either way. Thank you and kind regards | The effect sizes have been used depending on the test which was used for data.  For t-test, effect size was calculated using Cohen’s d (d) where d < 0.5 was small effect, 0.5 ≤ d < 0.8 was moderate effect, and d ≥ 0.8 was large effect. For Wilcoxon Signed Rank Test, effect size was calculated using Wilcoxon effect size (r) where r < 0.3 was small effect, 0.3 ≤ r < 0.5 was moderate effect, and r ≥ 0.5 was large effect. For ANOVA, generalized eta-squared (ƞ2) was used as effect size, where ƞ2 < 0.06 was small effect, 0.06 ≤ ƞ2 < 0.14 was medium effect, and ƞ2 ≥ 0.14 was large effect.  The same has been added in each table and also in the methodology section to make it better for the reader to understand. |
| Reviewer #3 | The article is well written. All technical comments were included in the revised version. A proper English review is requested before publication. | Thank you.  Amended and modified. |

: 
